# COVID-19 vaccination in the Gaza Strip: a cross-sectional study of vaccine coverage, hesitancy, and associated risk factors among community members and healthcare workers

**DOI:** 10.1186/s13031-022-00477-7

**Published:** 2022-09-09

**Authors:** Jennifer Majer, Jehad H. Elhissi, Nabil Mousa, Natalya Kostandova

**Affiliations:** 1 International Programs Department, International Medical Corps, Los Angeles, CA USA; 2 Department of Medicine, Al-Azhar University, Gaza City, Palestine; 3 Programs Department, International Medical Corps, Gaza City, Palestine; 4 Department of Epidemiology, Johns Hopkins Bloomberg School of Public Health, Baltimore, MD USA

**Keywords:** COVID-19, Vaccine, Hesitancy, Gaza Strip, Palestine, Humanitarian settings, Healthcare workers

## Abstract

**Background:**

The Gaza Strip, like other settings of complex humanitarian emergencies, faces immense challenges in vaccinating its population against COVID-19. This study was conducted in October 2021 among Gaza’s adult population and healthcare workers (HCWs). The primary aim was to estimate two indicators, coverage of COVID-19 vaccination and the prevalence of vaccine hesitancy. The secondary aim was to evaluate the two indicators’ associations with globally identified risk factors.

**Methods:**

A cross-sectional study was conducted using a population-based survey of adults and a purposive survey of HCWs in Gaza. A multi-stage sampling design was used for the population survey component. For the HCW component, five health facilities were purposively selected as entry points; HCWs in the facilities holding clinical or other specialized positions were approached to participate in the survey. Data were summarized as univariable descriptive statistics with unweighted and weighted point estimates. Logistic regression was used to evaluate associations of risk factors with vaccination status and vaccine hesitancy.

**Results:**

A total of 1075 individuals were surveyed, of whom 906 were community members and 169 were HCWs. Population-weighted vaccine coverage was estimated to be 49.08% (95% CI 43.10–55.08). 89.35% of HCWs were vaccinated. Population-weighted vaccine hesitancy was estimated to be 34.08% (95% CI 28.14–40.56) in the overall population and 67.24% (95% CI 49.04–81.41) among the unvaccinated sub-group. In logistic regression vaccination was independently associated with male sex (aOR 1.88, *p* = 0.006, 95% CI 1.20–2.95), older age (40+ vs. 18–39 age group) (aOR 1.92, *p* < 0.001, 95% CI 1.73–2.13), higher education (aOR 2.19, *p* < 0.001, 95% CI 1.51–3.17), and confidence in the safety of the vaccine (aOR 13.8, *p* < 0.001, 95% CI 10.1–18.8). Risk factors for hesitancy were similar to those identified for vaccination status, however hesitant individuals were somewhat more likely to obtain vaccine information from family members (aOR 1.29, *p* = 0.051, 95% CI 1.00–1.67) and less likely to trust healthcare providers (aOR 0.58, *p* < 0.001, 95% CI 0.49–0.68).

**Conclusions:**

The continued emergence of SARS-CoV-2 variants reinforces the importance of achieving high levels of vaccination coverage globally—a difficult undertaking in Gaza. This study estimated half of Gaza’s adult population received at least one dose of any COVID-19 vaccine by October 2021, and the majority of unvaccinated individuals were hesitant. Disparities in vaccination across the territory’s demographic groups underscore the need for targeted outreach to these populations and messaging through community-based channels to permeate social networks of the unvaccinated.

**Supplementary Information:**

The online version contains supplementary material available at 10.1186/s13031-022-00477-7.

## Background

The development of highly efficacious vaccines against the SARS-CoV-2 virus in 2020 was heralded as a potential end to the pandemic that has caused more than 6.39 million deaths globally as of August 2022 [1]. However even where vaccines were made widely available, roll-out progressed slowly or stagnated, in part due to persistent vaccine hesitancy [2, 3]. Vaccine hesitancy is defined by the World Health Organization (WHO) as “delay in acceptance or refusal of vaccination despite availability of vaccination services” [4]. Varying levels of vaccine hesitancy are reported globally; a review from February 2021 reported vaccine hesitancy across more than 30 assessed countries ranging from 10.0 to 57.8% [5, 6]. Among countries in the Middle East, data on vaccine hesitancy is sparse, or relies on data from web-based surveys that may exclude certain population groups, potentially resulting in biased estimates. A non-representative, web-based survey in Kuwait found vaccine hesitancy at 26% [7], while another online survey from December 2020 reported much higher levels of vaccine hesitancy—68.2% in Saudi Arabia, 71.6% in Jordan, and 76.4% in Kuwait [8]. Investigating and addressing the drivers of hesitancy may support efforts to increase uptake of the vaccine among populations who remain skeptical, misinformed, or fearful of vaccine risks. Surveys conducted to date have identified factors such as trust in government, sex, education level, risk perception, and use of social media for COVID-19 information, among others, to be associated with vaccine hesitancy [6, 8, 9].

Gaza Strip, the 362 sq km Palestinian territory bordering Israel and Egypt, faces immense challenges with vaccine roll-out given its fractured and poorly resourced health system and political obstacles to the importation of medical supplies [10, 11]. The first vaccine shipments arrived in Gaza in February 2021, however quantities were limited and directed to priority groups at risk of severe COVID-19 disease (older adults, people with cancer, and people with kidney disease) and healthcare workers [12]. In May 2021, a blockade of the territory during a two-week period of renewed conflict between the Israeli military and Hamas led to a temporary cessation of vaccine imports that further reduced availability [12]. By February 2022, the WHO estimated nearly 2 million people across the occupied Palestinian territory (oPt) had received at least one dose of the vaccine. The data also highlighted potential disparities in access to and uptake of the vaccine. While 44.82% of the Gazan population (aged 12 years and older) had been vaccinated and 32.51% were fully vaccinated, coverage was substantially higher in the West Bank, at 65.93% and 61.82% respectively [13].

Notably, vaccination levels have stagnated across the oPt in 2022 despite continued donations ensuring the availability of supplies [13]. Fewer than 100,000 people were newly vaccinated across the oPt between December 2021 and July 2022, compared to 1.91 million vaccinated in the previous 10 months [14]. The plateau suggests initial demand for vaccines was already saturated in 2021. A recent publication highlighted rumors, misinformation, and conspiracy theories as the main reasons for Palestinians’ “reluctance or unwillingness” to take the COVID-19 vaccine [12]. Limited data exist quantifying levels of vaccine hesitancy and its risk factors in Gaza; a web-based survey conducted in October 2020 found 63% of respondents would accept the vaccine (37% expressed hesitancy), with women and young adults less likely to be vaccine hesitant [11]. However to our knowledge no recent data was collected since the vaccination campaign began, and no population-representative study had been conducted.

The international non-governmental organization International Medical Corps (IMC) commissioned an external consultant epidemiologist to conduct a mixed methods vaccine hesitancy study in the Gaza Strip. The study was carried out between October 1 and 30, 2021 across all governorates in Gaza. The study aimed to investigate community members’ and healthcare workers’ perceptions of the COVID-19 vaccine; understand barriers and enablers associated with vaccine acceptance; and obtain updated estimates of COVID-19 vaccination coverage along with predictors of uptake. The results of the study provide new evidence on HCW and population perceptions that can inform COVID-19 service delivery and information campaigns in Gaza.

## Methods

The vaccine survey targeted two study populations—the general population of Gaza (community component) and a sub-population of HCWs (HCW component). For the community component of the survey, we carried out a population-based, cross-sectional survey of households across all five governorates. For the HCW component, a non-random survey design was used with a combination of purposive and convenience sampling.

### Survey instrument

A closed-ended questionnaire (see Additional file 1) was developed by an IMC expert working group in April 2021 based in part on the WHO recommended vaccine hesitancy assessment methodology [15]. Survey elements included current vaccination status, demographic characteristics, information sources, and perceived risks related to COVID-19. The questionnaire was translated into different languages by native speakers and standardized for organizational use, as the intention was to conduct vaccine hesitancy studies in different humanitarian contexts to draw cross-country comparisons. The Arabic version of the questionnaire was successfully field-tested in comparable settings, including Lebanon and Iraq, prior to Gaza.

### Sample size

Based on Gaza’s population demographics (1.99 million residents in 2019), we estimated the study population size to be 1,034,800 adults 18 years and older [16]. The sample size was calculated using EpiInfo (CDC) (v7.2.4.0) for a population survey with parameters set at 95% level of confidence, 50% estimated proportion, 5% margin of error, and a design effect of 2. Given lack of data from previous population surveys in Gaza, a conservative non-response rate of 15% was added. The resulting sample size was 884 households, while 906 were actually surveyed. For the HCW component, we targeted a sample size of between 30 and 40 HCWs from each facility based on the number of eligible staff, study timeline, and anticipated homogeneity of responses. 169 HCWs were ultimately surveyed.

### Sampling for population survey

Multi-stage stratified sampling was used to select households for the community component of the study (see Additional file 2). First, we compiled a list of geo-locations^1^ and the type of location (urban, rural, and refugee camp) for all five Gaza Strip Governorates (North, Gaza, Middle, Khan Yunis, and Rafah) using administrative data. From this list, we used stratified random sampling to select two geo-localities from each of the three strata (urban, refugee camps, and rural). We selected two geo-localities from each stratum largely due to insecurity and access constraints in Gaza; this approach reduced the number of areas data collection teams visited while maximizing the representativeness of the geographic types included in the study. In the second stage of sampling, we used simple random sampling to select four clusters (administrative units comprised of blocks in the camp settings and neighborhoods in the urban and rural areas) from each geo-location for a total of 24 clusters. Population data was not available at the cluster level in Gaza, so we calculated the number of households to be sampled in each cluster using probability proportional to size of governorates from which the clusters were selected [16] (see Additional file 3). Sampling weights are used in analysis to partially correct for population differences at the level of the geo-locality.

At the third stage, systematic random sampling was used to select households in each identified cluster. We used population data on households from the Palestinian Central Bureau of Statistics (PCBS) to estimate the total number of sampling units in the selected clusters and to calculate the sampling interval. Sampled households were replaced if the house was unoccupied. Enumerators approached the household selected for the survey and generally interviewed the first point of contact who was available (at home), willing (consented), and eligible (adult 18 years or older) to participate. In households where multiple individuals met these criteria and someone other than the first point of contact expressed a preference to participate, that person was interviewed instead. The targeting of adults through convenience sampling within households was largely based on time and resource constraints. Given a fixed number of days for data collection, enumerators could not consider household members who were not present.

### Sampling for HCW survey

For the HCW component of the study, five health care facilities were purposively selected as entry points: Caritas Gaza Health Center, Ahli Arab Hospital, Saint John’s Eye Hospital, Karama Hospital, and European Gaza Hospital. Four of the facilities had existing agreements with IMC to provide different levels of healthcare. The only facility not supported by IMC, European Gaza Hospital, is a public hospital mandated to receive COVID-19 patients experiencing moderate to severe symptoms that require in-patient care. Selection of these health facilities was primarily due to operational constraints in Gaza. Bi-lateral institutions that fund humanitarian aid in Gaza generally prohibit international non-governmental organizations from engaging with public sector service providers under the de-facto administration, as well as with non-vetted non-profit and private sector service providers. As a result, accessing health facilities outside of existing partnership networks poses certain risks, so we prioritized facilities already receiving technical and financial support from IMC.

Respondents were recruited to participate in the survey through a combination of purposive and convenience sampling. The study aimed to include a broad cadre of HCWs holding technical or clinical positions and commonly interfacing with patients. First, IMC enumerators consulted administrators at the five selected facilities to identity eligible staff (purposive component). Respondents were considered eligible HCWs if they held a position as either a medical doctor, nurse, dentist, midwife, pharmacist, lab technician, radiologist, or physiotherapist. A total of 655 staff were eligible. Second, facility administrators contacted eligible HCWs to describe the survey and request voluntary participation. HCWs who were available and willing to be surveyed were contacted by IMC enumerators to schedule interviews (convenience component).

Ultimately one-quarter (25.8%) of eligible staff were reached in the survey. The final sample consisted of 169 HCWs—17 HCWs from Caritas Gaza Health Center, 26 from Ahli Arab Hospital, 20 from Saint John’s Eye Hospital, 20 from Karama Hospital, and 86 from European Gaza Hospital.

### Data collection

Ten survey enumerators (5 men, 5 women) were recruited to conduct the survey. They were separated into five teams, with each team comprised of one female and one male enumerator to ensure culturally appropriate interviews could be conducted with women and men in the communities. The recruited enumerators were all young adults holding at least a bachelor’s degree and who had prior experience with data collection in Gaza. The research consultant provided a one-day training to enumerators on the data collection tool, interview techniques, sampling protocols, and COVID-19 prevention measures. Enumerators were also trained to phrase the survey question on vaccine hesitancy in a uniform way in Arabic based on local availability of the vaccine.

The survey was deployed using the electronic data collection application KoBoCollect (UNOCHA). Enumerators used tablets to collect and submit data on a daily basis. The teams were required to adhere to COVID-19 preventive measures recommended by the WHO and IMC (physical distancing, mask-wearing, and hand hygiene) during data collection. Informed consent was sought from all survey participants using standard formats. As data were recorded electronically and to avoid separate paper records, enumerators obtained consent orally rather than in writing.

Two staff members from IMC’s monitoring, evaluation, accountability and learning (MEAL) department supervised all data collection in the field, providing daily hands-on support and data quality assurance. The survey consultant also followed up regularly during data collection to ensure quality and integrity of the data.

### Data analysis

Table 1 shows the variables considered in the analysis. The primary outcomes used in the analysis were “COVID-19 vaccination status” and “COVID-19 vaccine hesitancy”. Individuals were classified as vaccinated if they responded “yes” to the question, “Have you already received COVID-19 vaccine?”, with at least one dose of any vaccine considered vaccinated.

**Table 1:** Thematic areas investigated in analysis

Primary outcome measures and determinants	Variables
Primary outcome: vaccination status	Vaccinated: defined as an adult member of the household 18 years or older who received at least one dose of any COVID-19 vaccine prior to the survey
Primary outcome: vaccine hesitancy	Vaccine hesitant: defined as lack of intent to receive a COVID-19 vaccine^a^
Demographic variables	Sex
	Age
	Governorate
	Highest level of education
	Health care worker versus community member
Information sources and needs	Perception of adequate information on COVID-19 vaccine
	Information needs (eligibility, timeline, side effects, effectiveness, registration)
	Current information sources (HCWs, CHWs, radio, television, newspapers, social media, friends and colleagues, local leaders, religious leaders, civil society organizations)
	Trusted sources (HCWs, CHWs, local leaders, religious leaders, family, neighbors and relatives, organizations, media, radio, television)
	Use of social media and types
	Sharing information on social media
Perceived risk	Self-perceived risk to get COVID-19 infection
	Self-perceived risk to develop severe disease following COVID-19 infection
	Perception of safety of the COVID-19 vaccine
	Past event with a vaccine dissuades from receiving the vaccine
	Knowledge of someone with severe outcome from not being vaccinated
	Preference for herd immunity/natural immunity
	Belief in better ways to prevent COVID-19 than the vaccine

*HCWs* healthcare workers, *CHWs* community health workers

^a^This survey item was operationalized through the question, “If you could get a COVID-19 vaccine this week, would you get it?” based on the recommended SAGE questionnaire. This question had three possible responses (yes, no, unsure). In the primary analysis, the vaccinated and unvaccinated population were pooled and respondents were classified as “vaccine hesitant” if they met both of two criteria: (1) did not previously receive the vaccine and (2) answered “no” or “unsure” to the question. the In sub-analysis, the same response categories were used to classify hesitancy, but data were restricted to the study’s non-vaccinated population

Vaccine hesitancy was classified in two ways for primary analysis and sub-analysis. For the primary analysis, vaccinated and unvaccinated respondents were pooled to increase statistical power, and vaccinated individuals were classified as non-hesitant. Individuals who were not vaccinated were classified as hesitant based on their response to the question “If the vaccine was available, would you get it?” according to the SAGE Working Group definition of vaccine hesitancy [4]. Unvaccinated individuals who responded “no” or “don’t know” to the question were classified as hesitant. In sub-analysis, data were restricted to the unvaccinated population, and risk factors for vaccine hesitancy were analysed within this sub-group. Hesitancy among unvaccinated individuals was classified using the same criteria as in the primary analysis.

Analyses were conducted using R version 4.0.5 [17] and RStudio version 1.4.1106 [18] with the packages *survey* [19] for all statistics and *gtsummary* [20] for the tables. All statistics described in the Results section represent population-weighted estimates. Data were weighted to the population using 2017 administrative data provided to IMC by the Palestinian Central Bureau of Statistics, using cluster sampling weights and household weights, and to account for stratification by type of geo-location (rural, urban, and refugee camp). As population in each cluster was not available, cluster weights within each selected geo-location were calculated using average cluster population in the selected geo-location. See Additional file 3 for the population of geo-localities. See Additional file 4 for details on finite population correction and sampling weights.

Demographic characteristics of the survey respondents are presented in Table 2. Demographic characteristics are presented as both unweighted and weighted univariable statistics, stratified by vaccination status (Table 3) and vaccine hesitancy status (Table 4). Univariable descriptive statistics with both unweighted and weighted results are presented for all other variables in Tables 5 (vaccination status) and 6 (vaccine hesitancy). Distributions of demographic characteristics and risk factor variables were compared using chi-square test with Rao & Scott's second-order correction.

**Table 2:** Demographic characteristics of survey respondents

Characteristic	Overall, N = 1075^a^	Community member, N = 906	HCW, N = 169
Age, n (%)			
18–39	659 (61.30)	527 (58.17)	132 (78.11)
40+	416 (38.70)	379 (41.83)	37 (21.89)
Geo-location, n (%)			
Gaza City	313 (29.12)	313 (34.55)	0 (0.00)
Healthcare Facility	169 (15.72)	0 (0.00)	169 (100.00)
Jabalia	190 (17.67)	190 (20.97)	0 (0.00)
Khanyunis	173 (16.09)	173 (19.09)	0 (0.00)
Nuseirat	133 (12.37)	133 (14.68)	0 (0.00)
Rafah	97 (9.02)	97 (10.71)	0 (0.00)
Strata, n (%)			
Camp	323 (30.05)	323 (35.65)	0 (0.00)
Healthcare Facility	168 (15.63)	0 (0.00)	168 (99.41)
Rural	163 (15.16)	162 (17.88)	1 (0.59)
Urban	421 (39.16)	421 (46.47)	0 (0.00)
Sex, n (%)			
Female	480 (44.65)	408 (45.03)	72 (42.60)
Male	595 (55.35)	498 (54.97)	97 (57.40)
Highest education level, n (%)			
Primary or none	83 (7.72)	81 (8.94)	2 (1.18)
Secondary or College/Vocational	353 (32.84)	347 (38.30)	6 (3.55)
University	639 (59.44)	478 (52.76)	161 (95.27)

^a^n (%)

**Table 3:** Demographic characteristics by vaccination status, community members only

Characteristic	Received vaccine (unweighted)			Received vaccine (weighted)		
	No, N = 451^a^	Yes, N = 455^a^	*p* value^b^	No (%)	Yes (%)	*p* value^b^
Age			**0.008**			**0.032**
18–39	282 (53.51%)	245 (46.49%)		53.83	46.17	
40+	169 (44.59%)	210 (55.41%)		47.04	52.96	
Sex			**< 0.001**			**0.018**
Female	238 (58.33%)	170 (41.67%)		58.55	41.45	
Male	213 (42.77%)	285 (57.23%)		45.13	54.87	
Highest education level			**< 0.001**			**0.007**
Primary or none	49 (60.49%)	32 (39.51%)		62.70	37.30	
Secondary or College/Vocational	194 (55.91%)	153 (44.09%)		56.88	43.12	
University	208 (43.51%)	270 (56.49%)		44.25	55.75	
Governorate			0.66			
Gaza City	159 (50.80%)	154 (49.20%)				
Jabalia	101 (53.16%)	89 (46.84%)				
Khanyunis	80 (46.24%)	93 (53.76%)				
Nuseirat	62 (46.62%)	71 (53.38%)				
Rafah	49 (50.52%)	48 (49.48%)				
Strata			0.12			
Camp	163 (50.46%)	160 (49.54%)				
Rural	69 (42.59%)	93 (57.41%)				
Urban	219 (52.02%)	202 (47.98%)				

Values in bold indicate statistically significant results at *p* value < 0.05

^a^n (%)

^b^Chi-squared test with Rao & Scott's second-order correction

**Table 4:** Demographic characteristics by vaccine hesitancy status, community members only

Characteristic	Vaccine hesitant (unweighted)			Vaccine hesitant (weighted)		
	No, N = 604^a^	Yes, N = 300^a^	*p* value^b^	No (%)	Yes (%)	*p* value^b^
Age			0.075			0.22
18–39	339 (64.45%)	187 (35.55%)		63.15	36.85	
40+	265 (70.11%)	113 (29.89%)		69.63	30.37	
Sex			0.082			**0.006**
Female	259 (63.79%)	147 (36.21%)		60.68	39.32	
Male	345 (69.28%)	153 (30.72%)		69.90	30.10	
Highest education level			**0.001**			0.18
Primary or none	40 (50.00%)	40 (50.00%)		46.80	53.20	
Secondary or College/Vocational	227 (65.42%)	120 (34.58%)		63.26	36.74	
University	337 (70.65%)	140 (29.35%)		71.58	28.42	
Governorate			0.060			
Gaza City	199 (63.58%)	114 (36.42%)				
Jabalia	124 (65.96%)	64 (34.04%)				
Khanyunis	132 (76.30%)	41 (23.70%)				
Nuseirat	87 (65.41%)	46 (34.59%)				
Rafah	62 (63.92%)	35 (36.08%)				
Strata			0.14			
Camp	211 (65.73%)	110 (34.27%)				
Rural	119 (73.46%)	43 (26.54%)				
Urban	274 (65.08%)	147 (34.92%)				

Values in bold indicate statistically significant results at *p* value < 0.05

^a^n (%)

^b^Chi-squared test with Rao & Scott's second-order correction

**Table 5:** Knowledge, beliefs, and other factors associated with vaccination status, community members only

Characteristic	N	Received vaccine (unweighted)			Received vaccine (weighted)		
		No, N = 451 (%)	Yes, N = 455 (%)	*p* value^a^	No, N = 715,034 (%)	Yes, N = 689,137 (%)	*p* value^a^
Vaccine hesitant	904	66.82	0.00	**< 0.001**	66.93	NA	**0.004**
Type of vaccine received	455			**< 0.001**			**0.002**
AstraZeneca		NA	0.66		NA	1.42	
Moderna		NA	17.80		NA	19.91	
Pfizer		NA	43.52		NA	40.69	
Sinopharm		NA	2.42		NA	3.04	
Sputnik		NA	35.16		NA	34.84	
Unsure		NA	0.44		NA	0.10	
Have enough information about COVID-19 vaccine	906			**< 0.001**			**0.009**
Yes		29.05	50.99		31.30	54.62	
No or don't know		70.95	49.01		68.70	45.38	
Information needed	905						
Eligibility criteria		35.70	31.72	0.21	51.46	53.24	0.84
Timeline for vaccine roll-out		7.76	6.39	0.42	13.80	11.17	0.18
Knowing when it's my turn to get the vaccine		8.65	3.08	**< 0.001**	12.34	5.63	**0.007**
Doses needed		21.73	16.96	0.070	27.64	21.53	0.057
Risks and side effects		89.14	80.84	**< 0.001**	90.57	85.06	0.22
Effectiveness of the vaccines		74.94	74.01	0.75	82.28	82.00	0.92
How to register for the vaccine		11.53	3.96	**< 0.001**	11.82	7.51	**0.038**
Common source of information about health and vaccines	904						
Health care providers		58.00	70.26	**< 0.001**	55.83	65.31	0.21
Community health care workers		20.89	25.99	0.071	13.18	16.33	0.26
Radio		14.22	10.35	0.077	13.80	13.03	0.67
Television		31.33	28.19	0.30	32.92	28.55	0.38
Newspapers		2.22	2.86	0.54	1.13	1.91	0.072
Mass events		10.00	3.96	**< 0.001**	9.15	2.45	**0.014**
Family		28.44	19.60	**0.002**	37.18	30.26	0.12
Neighbors, friends, colleagues		40.67	27.97	**< 0.001**	49.73	35.40	**0.008**
Local leaders		2.00	3.08	0.30	1.87	3.34	0.27
Religious leaders		2.67	5.29	**0.044**	3.59	7.08	**0.028**
Social media		60.44	56.39	0.22	61.33	61.64	0.95
Organizations		10.22	22.47	**< 0.001**	12.13	24.47	0.10
Trusted source of information	906						
Health care providers		62.53	75.38	**< 0.001**	62.60	72.08	0.053
Community health care workers		23.28	26.59	0.25	24.94	28.99	0.19
Local leaders		0.89	0.44	0.41	0.44	0.40	0.96
Religious leaders		1.11	1.32	0.77	0.84	0.83	0.99
Family members		5.54	3.74	0.20	7.33	5.92	0.24
Neighbors, friends, colleagues		7.10	3.52	**0.016**	9.41	3.72	0.10
Organizations		11.31	21.10	**< 0.001**	11.25	20.62	0.067
Media		16.85	8.57	**< 0.001**	16.37	10.28	0.17
Radio		2.44	3.08	0.56	3.42	1.93	0.067
Television		10.42	8.57	0.34	12.31	9.44	0.31
Other		7.54	5.27	0.16	6.18	5.48	0.61
Trust health providers and CHWs to provide with accurate information about the COVID-19 vaccine	903			**< 0.001**			**0.007**
No or don't know		17.56	11.26		19.29	11.91	
Yes		49.11	65.12		45.18	60.33	
Somewhat		33.33	23.62		35.53	27.76	
Follow social media platforms to get info about vaccine	906			> 0.99			0.81
No or don't know		24.39	24.40		20.28	19.28	
Yes		75.61	75.60		79.72	80.72	
Social media platforms used to get info about vaccine	685						
Facebook		99.12	97.09	0.052	99.84	98.33	0.057
Twitter		15.54	20.35	0.10	13.70	22.45	0.057
Instagram		39.59	36.63	0.43	45.96	45.26	0.87
YouTube		22.58	24.71	0.51	20.70	23.95	0.14
Shares information related to vaccine on social media network	863	14.66	25.23	**< 0.001**	10.56	20.58	0.061
How do you know info about vaccine from social media is true	906			**< 0.001**			**0.003**
I do not verify		34.59	18.68		40.26	18.96	
Other		16.41	13.19		14.75	15.78	
Verify from reputable website or health care provider		49.00	68.13		44.98	65.26	
Do leaders (religious, political, teachers, health care workers) in your community support the COVID-19 vaccines?	525			0.061			0.62
No or don't know		29.96	22.76		29.09	27.26	
Yes		70.04	77.24		70.91	72.74	
Most people you know interested in getting the COVID-19 vaccine	906			**< 0.001**			**< 0.001**
No or don't know		37.47	19.78		38.12	18.12	
Yes		22.84	44.84		23.38	47.36	
Somewhat		39.69	35.38		38.50	34.52	
How many people in your community are concerned about COVID-19 in the community	691			**0.022**			**0.015**
Few or some people, less than half		21.51	19.82		26.57	28.96	
Most people		36.59	28.23		29.59	15.69	
Some people—more than half		41.90	51.95		43.84	55.36	
COVID-19 vaccines will or are being rolled out equitably in community	902			0.056			0.17
No or don't know		22.05	17.00		24.89	21.93	
Yes		77.95	83.00		75.11	78.07	
Knows somebody that had a serious negative reaction to a vaccine that makes them reluctant to get COVID-19 vaccine	905			**< 0.001**			0.052
Yes		37.69	25.99		39.81	27.37	
Somewhat		13.97	11.45		19.65	17.30	
No or Don't know		48.34	62.56		40.54	55.34	
Consider COVID-19 vaccines safe	905			**< 0.001**			**0.001**
No or don't know		65.33	23.74		65.82	20.21	
Yes		8.00	37.58		7.33	34.76	
Somewhat		26.67	38.68		26.85	45.03	
Concerned about risks or side effects with COVID-19 vaccines	906			**< 0.001**			**< 0.001**
No or don't know		10.42	36.04		11.71	44.56	
Yes		79.16	38.24		79.35	32.14	
Somewhat		10.42	25.71		8.93	23.30	
Type of risks or side effects concerned about	693						
Fever		53.60	54.14	0.89	58.20	54.86	0.56
Body aches		62.78	60.00	0.46	68.28	61.44	0.28
Infertility		26.80	20.00	**0.039**	30.55	24.48	0.11
Physical disability		30.27	17.93	**< 0.001**	27.80	12.88	**0.007**
Death		60.30	35.52	**< 0.001**	59.20	36.01	**0.035**
Other		16.63	13.10	0.20	22.75	17.73	0.15
Type of COVID vaccine preferred	905			**< 0.001**			**0.001**
Other		0.00	0.22		0.00	0.01	
No preference		37.78	15.82		41.86	17.75	
Pfizer		33.56	47.47		33.42	43.90	
Sputnik		16.22	25.93		15.67	28.45	
Moderna		1.11	6.59		1.12	7.20	
Unsure		9.78	3.08		6.49	0.99	
Sinopharm		1.56	0.88		1.43	1.69	
Believe there are other (better) ways to prevent COVID-19 instead of vaccine	906			**< 0.001**			**0.033**
No or don't know		20.84	48.13		19.11	51.53	
Yes		65.41	33.63		63.30	30.06	
Somewhat		13.75	18.24		17.59	18.41	
Better ways to prevent COVID-19 instead of vaccine	448						
Social distance		83.05	86.27	0.38	83.97	87.96	0.49
Handwashing		76.61	79.08	0.55	79.04	78.38	0.82
Infection prevention and control		45.42	42.48	0.55	54.40	45.52	0.15
Ventilation		47.46	44.44	0.54	56.17	47.86	0.060
Wearing face masks		76.61	81.70	0.22	76.99	82.66	0.10
Know any person with a serious disease/disability that happened because they were not vaccinated	905			0.25			0.66
Yes		13.75	16.52		15.84	18.39	
No or don't know		86.25	83.48		84.16	81.61	
Think it is better to get COVID-19 and develop natural immunity than to get the vaccine	905			**< 0.001**			**< 0.001**
No		25.56	60.88		24.52	58.44	
Don't know		8.89	6.15		5.88	5.43	
Yes		51.56	18.46		53.85	19.88	
Somewhat		14.00	14.51		15.75	16.25	
Remember past events that would discourage them from getting COVID-19 vaccine	451	35.92	NA	**< 0.001**	39.58	NA	0.22
Think you are at risk to get COVID-19				0.36			0.58
No		8.43	11.23		9.37	9.19	
Don't know		7.76	7.71		7.90	9.29	
Yes		83.81	81.06		82.74	81.51	
Think you can get seriously ill, hospitalize or die if you get COVID-19	905			**0.034**			0.78
No		36.36	44.84		39.57	42.03	
Don't know		33.70	28.57		31.40	29.33	
Yes		29.93	26.59		29.03	28.64	
Barriers to receiving COVID-19 vaccine	450						
Availability		3.11	NA	**< 0.001**	2.13	NA	**0.001**
Distance to vaccination point		5.78	NA	**< 0.001**	1.97	NA	**< 0.001**
Cost		0.00	NA		0.00	NA	
Not a priority group		6.67	NA	**< 0.001**	5.29	NA	**0.001**
Lack of information about how/where to get it		21.78	NA	**< 0.001**	21.16	NA	0.27
Too stressful		36.44	NA	**< 0.001**	49.12	NA	0.94
Staff attitude		3.33	NA	**< 0.001**	3.34	NA	** < 0.001**
Not socially acceptable		28.00	NA	**< 0.001**	24.50	NA	**0.019**
None		10.89	NA	**< 0.001**	15.06	NA	**0.002**
Would get/have gotten the vaccine if employer recommended	451			**< 0.001**			**0.030**
No		35.48	NA		35.01	NA	
Unsure		15.30	NA		15.16	NA	
Yes		49.22	NA		49.83	NA	

Values in bold indicate statistically significant results at *p* value < 0.05

^a^Chi-squared test with Rao & Scott's second-order correction

**Table 6:** Knowledge, beliefs and other factors associated with vaccine hesitancy, community members only

Characteristic	N	Vaccine hesitant (unweighted)			Vaccine hesitant (weighted)		
		No, N = 604 (%)	Yes, N = 300 (%)	*p* value^a^	No, N = 925,538 (%)	Yes, N = 478,414 (%)	*p* value^a^
Received vaccine	904	75.33	0.00	**< 0.001**	74.46	NA	**0.004**
Type of vaccine received	455			**< 0.001**			**0.002**
AstraZeneca		0.66	NA		1.42	NA	
Moderna		17.80	NA		19.91	NA	
Pfizer		43.52	NA		40.69	NA	
Sinopharm		2.42	NA		3.04	NA	
Sputnik		35.16	NA		34.84	NA	
Unsure		0.44	NA		0.10	NA	
Have enough information about COVID-19 vaccine	904			**< 0.001**			**0.041**
Yes		46.03	28.33		50.15	28.44	
No or don't know		53.97	71.67		49.85	71.56	
Information needed	903						
Eligibility criteria		32.17	37.00	0.15	51.51	53.95	0.85
Timeline for vaccine roll-out		6.80	7.67	0.63	12.48	12.58	0.96
Knowing when it's my turn to get the vaccine		4.64	8.33	**0.027**	7.64	11.77	**0.035**
Doses needed		17.58	23.00	0.053	22.15	29.47	0.094
Risks and side effects		83.25	88.33	**0.045**	87.36	88.84	0.77
Effectiveness of the vaccines		74.63	74.33	0.92	82.82	80.86	0.30
How to register for the vaccine		5.64	12.00	**< 0.001**	8.31	12.40	0.052
Common source of information about health and vaccines	902						
Health care providers		67.66	57.53	**0.003**	64.21	53.29	0.062
Community health care workers		24.71	20.74	0.19	15.29	13.62	0.44
Radio		10.28	16.39	**0.009**	11.82	16.54	0.10
Television		28.19	33.11	0.13	28.23	35.71	0.42
Newspapers		2.32	3.01	0.54	1.43	1.67	0.45
Mass events		5.47	9.36	**0.029**	4.19	9.06	0.10
Family		21.23	29.10	**0.009**	29.76	41.55	**0.029**
Neighbors, friends, colleagues		29.68	43.48	**< 0.001**	37.63	52.52	0.070
Local leaders		2.99	1.67	0.24	3.27	1.26	0.42
Religious leaders		4.64	2.68	0.16	6.34	3.31	0.11
Social media		59.37	56.52	0.41	63.19	58.18	0.12
Organizations		20.40	8.36	**< 0.001**	22.20	10.42	**0.042**
Trusted source of information	904						
Health care providers		74.34	58.33	**< 0.001**	71.91	58.25	**0.006**
Community health care workers		25.83	23.33	0.42	27.23	26.35	0.82
Local leaders		0.83	0.33	0.39	0.63	0.02	**0.012**
Religious leaders		0.99	1.67	0.39	0.61	1.25	0.53
Family members		3.31	7.33	**0.007**	5.39	9.07	**0.039**
Neighbors, friends, colleagues		3.48	9.00	**< 0.001**	4.10	11.51	0.065
Organizations		19.54	9.67	**< 0.001**	19.21	9.34	**0.007**
Media		10.10	18.00	**< 0.001**	11.55	16.93	**0.035**
Radio		2.65	3.00	0.76	1.66	4.66	**0.010**
Television		8.77	11.00	0.28	9.47	13.68	0.43
Other		5.13	8.67	**0.040**	4.85	7.74	0.058
Trust health providers and CHWs to provide with accurate information about the COVID-19 vaccine	901			**< 0.001**			**0.005**
No or don't know		9.80	23.75		10.63	25.45	
Yes		66.61	38.13		62.07	34.26	
Somewhat		23.59	38.13		27.30	40.29	
Follow social media platforms to get info about vaccine	904			0.35			0.16
No or don't know		23.51	26.33		18.44	22.40	
Yes		76.49	73.67		81.56	77.60	
Social media platforms used to get info about vaccine	683						
Facebook		97.62	99.10	0.19	98.69	99.93	**0.015**
Twitter		18.61	16.74	0.55	20.50	13.01	0.19
Instagram		37.88	38.91	0.79	44.86	47.18	0.63
YouTube		22.51	26.24	0.28	22.41	22.12	0.93
Shares information related to vaccine on social media network	861	23.45	13.17	**< 0.001**	19.72	7.32	**0.014**
How do you know info about vaccine from social media is true	904			**< 0.001**			**0.006**
I do not verify		19.04	41.67		20.67	47.49	
Other		13.41	17.67		15.13	15.51	
Verify from reputable website or health care provider		67.55	40.67		64.20	37.01	
Do leaders (religious, political, teachers, health care workers) in your community support the COVID-19 vaccines?	525			**0.009**			0.24
No or don't know		22.93	33.74		26.10	32.82	
Yes		77.07	66.26		73.90	67.18	
Most people you know interested in getting the COVID-19 vaccine	904			**< 0.001**			**0.017**
No or don't know		20.53	45.00		20.48	43.45	
Yes		43.38	14.67		45.64	14.84	
Somewhat		36.09	40.33		33.88	41.71	
How many people in your community are concerned about COVID-19 in the community	689			**0.024**			0.35
Few or some people, less than half		17.78	26.36		25.57	31.84	
Most people		32.89	31.80		21.55	25.22	
Some people—more than half		49.33	41.84		52.88	42.94	
COVID-19 vaccines will or are being rolled out equitably in community	900			**0.023**			0.27
No or don't know		17.44	23.83		22.19	25.87	
Yes		82.56	76.17		77.81	74.13	
Knows somebody that had a serious negative reaction to a vaccine that makes them reluctant to get COVID-19 vaccine	903			**< 0.001**			0.17
Yes		27.36	40.67		29.41	42.00	
Somewhat		11.61	15.00		16.56	22.25	
No or don't know		61.03	44.33		54.03	35.74	
Consider COVID-19 vaccines safe	903			**< 0.001**			**0.007**
No or don't know		28.48	76.25		27.31	74.61	
Yes		32.62	3.34		30.34	2.33	
Somewhat		38.91	20.40		42.35	23.07	
Concerned about risks or side effects with COVID-19 vaccines	904			**< 0.001**			**0.003**
No or don't know		30.96	7.67		38.22	7.73	
Yes		46.36	83.33		41.80	84.02	
Somewhat		22.68	9.00		19.99	8.25	
Risks or side effects concerned about	692						
Fever		55.29	51.81	0.37	56.09	58.06	0.76
Body aches		62.02	61.23	0.83	64.78	66.92	0.49
Infertility		19.71	30.43	**0.001**	21.78	36.66	**0.014**
Physical disability		19.47	33.33	**< 0.001**	15.74	30.52	**0.009**
Death		39.66	65.58	**< 0.001**	39.59	64.59	0.073
Other		13.70	17.39	0.19	19.79	22.26	0.39
Type of COVID vaccine preferred	903			**< 0.001**			**0.004**
Other		0.17	0.00		0.01	0.00	
No preference		15.07	49.83		17.40	54.43	
Pfizer		48.01	25.75		44.62	26.85	
Sputnik		26.49	10.37		28.40	9.47	
Moderna		4.97	1.67		5.36	1.68	
Unsure		3.97	11.37		2.15	6.98	
Sinopharm		1.32	1.00		2.06	0.59	
Believe there are other (better) ways to prevent COVID-19 instead of vaccine	904			**< 0.001**			**0.016**
No or don't know		43.71	16.33		45.34	15.06	
Yes		38.58	71.33		36.06	68.13	
Somewhat		17.72	12.33		18.60	16.81	
Better ways to prevent COVID-19 instead of vaccine	447						
Social distance		85.84	82.24	0.30	87.05	83.35	0.25
Handwashing		78.97	75.70	0.41	77.05	80.65	0.35
Infection prevention and control		42.06	47.20	0.28	48.68	54.64	0.27
Ventilation		45.49	47.66	0.65	48.66	58.59	0.29
Wearing face masks		80.69	75.70	0.20	78.74	78.79	0.99
Know any person with a serious disease/disability that happened because they were NOT vaccinated	903			**0.008**			0.22
Yes		17.41	10.67		20.56	10.40	
No or don't know		82.59	89.33		79.44	89.60	
Think it is better to get COVID-19 and develop natural immunity than to get the vaccine	903			**< 0.001**			**0.005**
No		55.56	19.00		53.44	17.46	
Don't know		6.47	9.67		4.75	7.41	
Yes		23.55	57.33		26.17	58.43	
Somewhat		14.43	14.00		15.63	16.71	
Remember past events that would discourage them from getting COVID-19 vaccine	449			0.36			0.52
Yes		32.89	37.33		37.28	40.71	
No or don't know		67.11	62.67		62.72	59.29	
Think you are at risk to get COVID-19	903			0.11			0.30
No		9.62	10.33		8.88	10.07	
Don't know		6.47	10.33		7.16	11.33	
Yes		83.91	79.33		83.96	78.60	
Think you can get seriously ill, hospitalize or die if you get COVID-19	904			**0.014**			0.072
No		42.38	37.33		40.20	41.92	
Don't know		27.81	37.33		27.96	35.02	
Yes		29.80	25.33		31.84	23.05	
Barriers for receiving COVID-19 vaccine	448						
Availability		2.70	3.33	0.72	3.12	1.64	0.15
Distance to vaccination point		6.08	5.67	0.86	2.55	1.69	0.30
Cost		0.00	0.00		0.00	0.00	
Not a priority group		6.08	6.67	0.81	2.17	6.80	0.23
Lack of information about how/where to get it		20.95	22.33	0.74	22.63	20.45	0.57
Too stressful		35.14	37.33	0.65	45.28	51.03	0.10
Staff attitude		3.38	3.33	0.98	3.52	3.26	0.92
Not socially acceptable		11.49	36.33	**< 0.001**	11.22	31.03	0.063
None		10.81	11.00	0.95	17.80	13.73	0.37
Would get/have gotten the vaccine if employer recommended	449			**< 0.001**			0.089
No		19.46	43.33		22.32	41.27	
Unsure		6.71	19.33		4.27	20.53	
Yes		73.83	37.33		73.41	38.20	

Values in bold indicate statistically significant results at *p* value < 0.05

^a^Chi-squared test with Rao & Scott's second-order correction

Unadjusted odds ratios (ORs) were estimated using logistic regression (Tables 7, 8). Adjusted odds ratios (aORs) (Tables 9, 10) were estimated using multivariable logistic regression models, adjusted for sex, age, highest education received, and hypothesized risk factors based on literature, which  included having enough information on the vaccine, receiving information from healthcare workers, receiving information from family, receiving information from social media, trusting information from healthcare workers, perception of vaccine safety, and believing in personal risk of serious illness or hospitalization from COVID-19. For each, estimates of unadjusted and adjusted ORs, *p* values and 95% CI’s are presented; a *p* value of < 0.05 is considered significant.

**Table 7:** Unadjusted odds ratios of vaccine receipt, community members only

Characteristic	OR (95% CI)^a^	*p* value
Sex		**< 0.001**
Female	–	
Male	1.72 (1.37 to 2.16)	
Age		**< 0.001**
18–39	–	
40+	1.31 (1.14 to 1.51)	
Highest education level		**< 0.001**
Primary or none	–	
Secondary or College/Vocational	1.27 (1.03 to 1.57)	
University	2.12 (1.85 to 2.42)	
Have enough information about COVID-19 vaccine		**< 0.001**
Yes	–	
No or don't know	0.38 (0.28 to 0.52)	
Information needed		
Eligibility criteria	1.07 (0.56 to 2.05)	0.83
Timeline for vaccine roll-out	0.79 (0.60 to 1.04)	0.087
Knowing when it's my turn to get the vaccine	0.42 (0.33 to 0.55)	**< 0.001**
Doses needed	0.72 (0.58 to 0.89)	**0.003**
Risks and side effects	0.59 (0.30 to 1.15)	0.12
Effectiveness of the vaccines	0.98 (0.69 to 1.39)	0.91
How to register for the vaccine	0.61 (0.46 to 0.80)	**< 0.001**
Common source of information about health and vaccines		
Health care providers	1.49 (0.91 to 2.45)	0.12
Community health care workers	1.29 (0.90 to 1.84)	0.17
Radio	0.94 (0.70 to 1.24)	0.64
Television	0.81 (0.55 to 1.20)	0.30
Newspapers	1.71 (1.16 to 2.52)	**0.007**
Mass events	0.25 (0.14 to 0.44)	**< 0.001**
Family	0.73 (0.55 to 0.98)	**0.033**
Neighbors, friends, colleagues	0.55 (0.46 to 0.66)	**< 0.001**
Local leaders	1.82 (0.76 to 4.35)	0.18
Religious leaders	2.04 (1.43 to 2.92)	**< 0.001**
Social media	1.01 (0.70 to 1.47)	0.94
Organizations	2.35 (1.13 to 4.88)	**0.022**
Trusted source of information		
Health care providers	1.54 (1.17 to 2.03)	**0.002**
Community health care workers	1.23 (0.97 to 1.56)	0.088
Radio	0.56 (0.37 to 0.84)	**0.005**
Television	0.74 (0.46 to 1.19)	0.22
Family members	0.80 (0.59 to 1.08)	0.14
Neighbors, friends, colleagues	0.37 (0.16 to 0.87)	**0.022**
Local leaders	0.90 (0.03 to 28.6)	0.95
Religious leaders	0.98 (0.17 to 5.84)	0.99
Media	0.59 (0.32 to 1.06)	0.077
Organizations	2.05 (1.23 to 3.40)	**0.006**
Other	0.88 (0.57 to 1.36)	0.57
Follow social media platforms to get info about vaccine		0.79
No or don't know	–	
Yes	1.06 (0.67 to 1.68)	
Social media platforms used to get info about vaccine	0.09 (0.01 to 0.64)	**0.016**
Facebook	1.82 (1.23 to 2.70)	**0.003**
Twitter	0.97 (0.72 to 1.32)	0.85
Instagram	1.21 (1.00 to 1.45)	**0.046**
YouTube		**0.004**
Shares information related to vaccine on social media network	–	
No	2.20 (1.29 to 3.75)	
Yes		0.60
How do you know info about vaccine from social media is true		**< 0.001**
I do not verify	–	
Other	2.27 (1.87 to 2.76)	
Verify from reputable website or health care provider	3.08 (2.31 to 4.11)	
Think you can get seriously ill, hospitalized or die if you get COVID-19		**< 0.001**
No	–	
Don't know	0.88 (0.68 to 1.14)	
Yes	0.93 (0.50 to 1.73)	
Consider COVID-19 vaccines safe		**< 0.001**
No or don't know	–	
Yes	15.4 (12.2 to 19.6)	
Somewhat	5.46 (3.56 to 8.38)	

Values in bold indicate statistically significant results at *p* value < 0.05

^a^*OR* odds ratio, *CI* confidence interval

**Table 8:** Unadjusted odds ratios of vaccine hesitancy, community members only

Characteristic	OR (95% CI)^a^	*p* value
Sex		**< 0.001**
Female	–	
Male	0.66 (0.59 to 0.75)	
Age		0.12
18–39	–	
40+	0.75 (0.52 to 1.08)	
Highest education level		**< 0.001**
Primary or none	–	
Secondary or College/Vocational	0.51 (0.17 to 1.54)	
University	0.35 (0.21 to 0.59)	
Have enough information about COVID-19 vaccine		**< 0.001**
Yes	–	
No or don't know	2.53 (1.48 to 4.32)	
Information needed		
Eligibility criteria	1.10 (0.43 to 2.85)	0.84
Timeline for vaccine roll-out	1.01 (0.73 to 1.40)	0.95
Knowing when it's my turn to get the vaccine	1.61 (1.25 to 2.09)	**< 0.001**
Doses needed	1.47 (1.08 to 2.01)	**0.016**
Risks and side effects	1.15 (0.48 to 2.75)	0.75
Effectiveness of the vaccines	0.88 (0.71 to 1.08)	0.21
How to register for the vaccine	1.56 (1.18 to 2.07)	**0.002**
Common source of information about health and vaccines		
Health care providers	0.64 (0.47 to 0.86)	**0.004**
Community health care workers	0.87 (0.65 to 1.18)	0.37
Radio	1.48 (1.07 to 2.03)	**0.017**
Television	1.41 (0.68 to 2.93)	0.35
Newspapers	1.17 (0.82 to 1.67)	0.39
Mass events	2.28 (1.12 to 4.62)	**0.023**
Family	1.68 (1.29 to 2.17)	**< 0.001**
Neighbors, friends, colleagues	1.83 (1.19 to 2.82)	**0.006**
Local leaders	0.38 (0.04 to 3.19)	0.37
Religious leaders	0.51 (0.28 to 0.92)	**0.025**
Social media	0.81 (0.67 to 0.98)	**0.031**
Organizations	0.41 (0.24 to 0.69)	**< 0.001**
Trusted source of information		
Health care providers	0.54 (0.46 to 0.65)	**< 0.001**
Community health care workers	0.95 (0.67 to 1.35)	0.79
Radio	2.87 (1.99 to 4.14)	**< 0.001**
Television	1.52 (0.61 to 3.76)	0.37
Family members	1.75 (1.28 to 2.40)	**< 0.001**
Neighbors, friends, colleagues	3.05 (1.37 to 6.77)	**0.006**
Local leaders	0.02 (0.00 to 0.18)	**< 0.001**
Religious leaders	2.05 (0.26 to 16.0)	0.49
Media	1.56 (1.23 to 1.99)	**< 0.001**
Organizations	0.43 (0.34 to 0.56)	**< 0.001**
Other	1.65 (1.19 to 2.29)	**0.003**
Follow social media platforms to get info about vaccine		0.063
No or don't know	–	
Yes	0.78 (0.61 to 1.01)	
Social media platforms used to get info about vaccine		
Facebook (ref: no)	18.8 (3.81 to 92.8)	**< 0.001**
Twitter (ref: no)	0.58 (0.30 to 1.11)	0.10
Instagram (ref: no)	1.10 (0.78 to 1.55)	0.60
YouTube (ref: no)	0.98 (0.69 to 1.40)	0.93
Shares information related to vaccine on social media network		**< 0.001**
No	–	
Yes	0.32 (0.21 to 0.50)	
How do you know info about vaccine from social media is true		**< 0.001**
I do not verify	–	
Other	0.45 (0.34 to 0.58)	
Verify from reputable website or health care provider	0.25 (0.19 to 0.34)	
Think you can get seriously ill, hospitalized or die if you get COVID-19		**< 0.001**
No	–	
Don't know	1.20 (0.87 to 1.67)	
Yes	0.69 (0.50 to 0.97)	
Consider COVID-19 vaccines safe		**< 0.001**
No or Don't know	–	
Yes	0.03 (0.01 to 0.16)	
Somewhat	0.20 (0.17 to 0.23)	

Values in bold indicate statistically significant results at *p* value < 0.05

^a^*OR* odds ratio, *CI* confidence interval

**Table 9:** Adjusted odds ratios of vaccine receipt, community members only

Characteristic	OR (95% CI)^a^	*p* value
Sex		**0.006**
Female	–	
Male	1.88 (1.20 to 2.95)	
Age		**< 0.001**
18–39	–	
40+	1.92 (1.73 to 2.13)	
Highest education level		**< 0.001**
Primary or none	–	
Secondary or College/Vocational	1.39 (0.92 to 2.09)	
University	2.19 (1.51 to 3.17)	
Have enough information about COVID-19 vaccine		**< 0.001**
Yes	–	
No or don't know	0.62 (0.48 to 0.80)	
Common source of information about health and vaccines		
Health care providers (ref: no)	1.03 (0.50 to 2.14)	0.93
Family (ref: no)	1.11 (0.66 to 1.86)	0.70
Social media (ref: no)	0.86 (0.62 to 1.19)	0.37
Trust health care providers for information on COVID-19 vaccine	1.24 (1.00 to 1.54)	0.052
Consider COVID-19 vaccines safe		**< 0.001**
No or don't know	–	
Yes	13.8 (10.1 to 18.8)	
Somewhat	5.41 (3.69 to 7.94)	
Think you can get seriously ill, hospitalize or die if you get COVID-19		0.62
No	–	
Don't know	1.02 (0.78 to 1.32)	
Yes	0.86 (0.59 to 1.24)	

Values in bold indicate statistically significant results at *p* value < 0.05

^a^*OR* odds ratio, *CI* confidence interval

**Table 10:** Adjusted odds ratios of vaccine hesitancy, community members only

Characteristic	OR (95% CI)^a^	*p* value
Sex		**0.004**
Female	–	
Male	0.58 (0.40 to 0.84)	
Age		**0.005**
18–39	–	
40+	0.47 (0.28 to 0.80)	
Highest education level		**< 0.001**
Primary or none	–	
Secondary or College/Vocational	0.45 (0.22 to 0.93)	
University	0.32 (0.25 to 0.42)	
Have enough information about COVID-19 vaccine		0.11
Yes	–	
No or don't know	1.39 (0.93 to 2.07)	
Common source of information about health and vaccines		
Health care providers (ref: no)	1.01 (0.74 to 1.39)	0.93
Family (ref: no)	1.29 (1.00 to 1.67)	0.051
Social media (ref: no)	0.81 (0.60 to 1.10)	0.18
Trusted source of information: Health care providers	0.58 (0.49 to 0.68)	**< 0.001**
Think you can get seriously ill, hospitalized or die if you get COVID-19		**0.005**
No	–	
Don't know	1.08 (0.76 to 1.53)	
Yes	0.62 (0.37 to 1.04)	
Consider COVID-19 vaccines safe		**< 0.001**
No or don't know	–	
Yes	0.03 (0.01 to 0.15)	
Somewhat	0.21 (0.18 to 0.25)	

Values in bold indicate statistically significant results at *p* value < 0.05

^a^*OR* odds ratio, *CI* confidence interval

## Results

### Respondent characteristics

1075 people (906 community members and 169 health professionals) participated in the survey (Table 2). The demographic and geographic distribution of community members respondents generally mirrors the population of Gaza. Females represented 45.03% of respondents, compared to 49.3% of the global population of Palestinians estimated to be women and girls [21]. The largest proportion of respondents were from Gaza City (34.55%) and the lowest from Rafah (10.71%)*,* which is similar to the PCBS population projections for Gaza Strip where it is estimated 34% of the population reside in Gaza City and 12% in Rafah [22]. Younger people may be over-represented in our sample. 58.17% of respondents were between 18 and 39 years-old, while the PCBS estimated the 18–29 age group comprised just 21.8% of the population [23]. Although not a random sample, the HCWs surveyed resemble the demographics of the Palestinian health workforce. In our study 78.11% of HCW respondents were 18–39 years-old and 42.60% were female. A recent Palestinian healthcare labor mapping survey (2021) found 74% of HCWs were under 45 years-old, while the proportion of female healthworkers varied substantially according to the professional category but was generally balanced [24].

### Vaccination status and characteristics

Table 3 presents the unweighted and weighted distribution of the characteristics of the population by vaccination status. Table 5 presents the unweighted and weighted distribution of risk factors by vaccination status and Table 7 presents the unadjusted odds ratios. Population-weighted vaccination coverage was 49.08% (95% CI 43.10–55.08). Males were more likely to be vaccinated than females (54.87% vs. 41.45%, *p* = 0.018). Older adults (40 years and older) were more likely to be vaccinated than younger adults (52.96% vs. 46.17%, *p* = 0.032). There was an increasing trend in vaccination with higher levels of education; respondents with university degrees were twice as likely to be vaccinated compared to those with primary or no education (OR 2.12, 95% CI 1.85–2.42), and those with secondary or college/vocational education were 27% more likely to be vaccinated than those with primary or no formal education (OR 1.27, 95% CI 1.03–1.57).

Certain self-reported perceptions of the risks and trade-offs of COVID-19 vaccination were associated with vaccination status. The vaccinated had 15 times higher odds of considering the vaccines safe (OR 15.4, 95% CI 12.2–19.60). They were also less likely to express concerns about side effects and less likely to prefer natural immunity to the vaccine. The unvaccinated group reported concerns specifically about physical disability (27.80%) and death (59.20%) at higher levels than the unvaccinated. Concerns about fever and body aches were also common and reported by more than 50% of both the vaccinated and unvaccinated population.

More than 80% of both groups believed they were at risk of COVID-19 infection, while less than one-third believed they were at risk of severe illness and hospitalization (Table 5). This suggests perceptions of risk of infection are universally high; however, people do not largely believe they are at risk of developing serious disease, and these views do not appear to vary significantly among vaccinated and unvaccinated individuals (*p* > 0.05).

Perceptions of adequate vaccine information and the channels used to access information differed among the vaccinated and unvaccinated population. 54.62% of vaccinated individuals reported having enough information about the vaccine, while 31.30% of the unvaccinated believed they received sufficient information. Those who reported having inadequate information had around 60% lower odds of being vaccinated (OR 0.38, *p* < 0.001, 95% CI 0.28–0.52). Among both vaccinated and unvaccinated individuals, the most commonly requested information was on side effects and effectiveness of the vaccines, as reported by  more than 80% of both groups. Notably, slightly more than 11% of the unvaccinated expressed interest in information on how to register for the vaccine (Table 11).

**Table 11:** Factors associated with vaccine hesitancy, non-vaccinated community members only (n = 449)

Characteristic	N	Vaccine hesitant (unweighted)			Vaccine hesitant (weighted)		
		No, N = 149 (%)	Yes, N = 300 (%)	*p* value^a^	No (%)	Yes (%)	*p* value^a^
Age	449			0.88			0.41
18–39		63.09	62.33		56.69	62.58	
40+		36.91	37.67		43.31	37.42	
Sex	449			**0.033**			0.68
Female		59.73	49.00		48.40	49.89	
Male		40.27	51.00		51.60	50.11	
Highest education level	449			**0.018**			0.41
Primary or none		5.37	13.33		5.98	14.97	
Secondary or College/Vocational		49.66	40.00		44.79	41.85	
University		44.97	46.67		49.24	43.18	
Have enough information about COVID-19 vaccine	449			0.58			0.30
Yes		30.87	28.33		37.74	28.76	
No or don't know		69.13	71.67		62.26	71.24	
Information needed	449						
Eligibility criteria		33.56	37.00	0.47	46.59	53.75	0.58
Timeline for vaccine roll-out		8.05	7.67	0.89	17.02	12.82	0.35
When it's my turn to get the vaccine		9.40	8.33	0.71	14.14	12.06	0.59
Doses needed		19.46	23.00	0.39	25.10	30.53	0.50
Risks and side effects		90.60	88.33	0.47	94.12	89.80	0.12
Effectiveness of the vaccines		76.51	74.33	0.62	84.62	81.19	0.27
How to register for the vaccine		10.74	12.00	0.69	11.05	12.47	0.75
Common source of information about health and vaccines	448						
Health care providers		59.73	57.53	0.66	62.75	53.21	0.16
Community health care workers		20.81	20.74	0.99	12.33	13.66	0.54
Radio		10.07	16.39	0.073	8.61	16.61	0.067
Television		28.19	33.11	0.29	27.28	35.24	0.43
Newspapers		0.67	3.01	0.12	0.05	1.71	**0.018**
Mass events		10.07	9.36	0.81	9.16	9.03	0.97
Family		26.17	29.10	0.52	29.06	43.28	0.15
Neighbors, friends, colleagues		34.90	43.48	0.083	44.04	52.29	0.51
Local leaders		2.68	1.67	0.47	3.36	1.38	0.51
Religious leaders		2.68	2.68	> 0.99	4.55	3.41	0.63
Social media		68.46	56.52	**0.015**	66.73	58.32	0.058
Organizations		14.09	8.36	0.061	15.36	10.30	0.17
Trusted source of information	449						
Health care providers		71.14	58.33	**0.009**	73.03	58.60	0.14
Community health care workers		23.49	23.33	0.97	22.87	26.79	0.69
Local leaders		2.01	0.33	0.075	1.12	0.02	**0.012**
Religious leaders		0.00	1.67	0.11	0.00	1.20	0.35
Family members		2.01	7.33	**0.021**	4.05	9.51	0.075
Neighbors, friends, colleagues		3.36	9.00	**0.029**	4.72	10.81	0.082
Organizations		14.77	9.67	0.11	14.47	9.17	0.45
Media		14.77	18.00	0.39	13.40	15.69	0.40
Radio		1.34	3.00	0.29	0.82	4.70	**0.012**
Television		9.40	11.00	0.60	9.12	12.88	0.58
Other		4.70	8.67	0.13	2.81	7.78	0.069
Trust health providers and CHWs to provide with accurate information about the COVID-19 vaccine	448			**< 0.001**			**0.001**
No or don't know		5.37	23.75		6.66	25.71	
Yes		71.14	38.13		67.87	33.96	
Somewhat		23.49	38.13		25.48	40.33	
Follow social media platforms to get info about vaccine	449			0.20			0.15
No or don't know		20.81	26.33		15.69	22.34	
Yes		79.19	73.67		84.31	77.66	
Social media platforms used to get info about vaccine	339						
Facebook		99.15	99.10	0.96	99.67	99.93	0.25
Twitter		13.56	16.74	0.44	15.49	12.84	0.49
Instagram		41.53	38.91	0.64	44.09	47.97	0.78
YouTube		16.10	26.24	**0.034**	18.32	21.91	0.45
Shares information related to vaccine on social media network	421	17.86	13.17	0.20	16.82	6.94	**0.005**
How do you know info about vaccine from social media is true	449			**< 0.001**			0.16
I do not verify		20.13	41.67		24.76	46.56	
Other		14.09	17.67		13.72	15.54	
Verify from reputable website or health care provider		65.77	40.67		61.52	37.90	
Do leaders (religious, political, teachers, health care workers) in your community support the COVID-19 vaccines?	257			0.082			0.45
No or don't know		23.40	33.74		24.83	32.43	
Yes		76.60	66.26		75.17	67.57	
Most people you know interested in getting the COVID-19 vaccine	449			**< 0.001**			**0.036**
No or don't know		22.82	45.00		27.78	43.95	
Yes		38.93	14.67		39.38	14.48	
Somewhat		38.26	40.33		32.83	41.57	
How many people in your community are concerned about COVID-19 in the community	356			**0.003**			0.17
Few or some people, less than half		11.97	26.36		15.20	31.45	
Most people		46.15	31.80		40.77	26.16	
Some people—more than half		41.88	41.84		44.03	42.39	
COVID-19 vaccines will or are being rolled out equitably in community	447			0.23			0.63
No or don't know		18.79	23.83		23.28	26.78	
Yes		81.21	76.17		76.72	73.22	
Knows somebody that had a serious negative reaction to a vaccine that makes them reluctant to get COVID-19 vaccine	449			0.056			0.26
Yes		31.54	40.67		34.84	42.93	
Somewhat		12.08	15.00		14.85	21.93	
No or don't know		56.38	44.33		50.31	35.14	
Consider COVID-19 vaccines safe	448			**< 0.001**			0.074
No or don't know		42.95	76.25		49.16	74.25	
Yes		17.45	3.34		17.38	2.08	
Somewhat		39.60	20.40		33.46	23.67	
Concerned about risks or side effects with COVID-19 vaccines	449			**0.009**			0.14
No or don't know		15.44	7.67		19.21	7.48	
Yes		71.14	83.33		69.81	83.79	
Somewhat		13.42	9.00		10.97	8.73	
Types of risks or side effects concerned about	402						
Fever		57.94	51.81	0.25	58.26	58.56	0.95
Body aches		66.67	61.23	0.30	73.25	66.99	0.17
Infertility		19.05	30.43	**0.017**	16.85	36.95	0.068
Physical disability		23.02	33.33	**0.037**	23.34	31.23	0.090
Death		49.21	65.58	**0.002**	47.90	64.35	0.17
Other		15.08	17.39	0.56	23.16	21.99	0.74
Type of COVID vaccine preferred	448						
Other		0.00	0.00		0.00	0.00	
No preference		12.75	49.83		17.25	53.89	
Pfizer		49.66	25.75		47.01	27.47	
Sputnik		28.19	10.37		27.00	9.54	
Moderna		0.00	1.67		0.00	1.73	
Unsure		6.71	11.37		5.48	6.75	
Sinopharm		2.68	1.00		3.26	0.63	
Believe there are other (better) ways to prevent COVID-19 instead of vaccine	449			**< 0.001**			**0.039**
No or don't know		30.20	16.33		28.51	15.14	
Yes		53.69	71.33		51.93	67.67	
Somewhat		16.11	12.33		19.56	17.19	
Better ways to prevent COVID-19 instead of vaccine	294						
Social distance		85.00	82.24	0.58	86.75	84.33	0.67
Handwashing		78.75	75.70	0.58	75.60	81.53	0.45
Infection prevention and control		41.25	47.20	0.36	57.90	55.56	0.83
Ventilation		47.50	47.66	0.98	52.95	59.28	0.60
Wearing face masks		78.75	75.70	0.58	73.25	79.59	0.25
Know any person with a serious disease/disability that happened because they were NOT vaccinated	449			**0.006**			0.13
Yes		20.13	10.67		26.73	10.22	
No or don't know		79.87	89.33		73.27	89.78	
Think it is better to get COVID-19 and develop natural immunity than to get the vaccine	448			**< 0.001**			0.18
No		39.19	19.00		39.21	17.81	
Don't know		7.43	9.67		2.94	7.63	
Yes		39.19	57.33		43.45	57.73	
Somewhat		14.19	14.00		14.39	16.83	
Remember past events that would discourage them from getting COVID-19 vaccine	449	32.89	37.33	0.36	35.22	40.21	0.47
Think you are at risk to get COVID-19	449			**0.001**			0.22
No		4.70	10.33		7.73	9.80	
Don't know		2.68	10.33		0.89	11.49	
Yes		92.62	79.33		91.38	78.72	
Think you can get seriously ill, hospitalized or die if you get COVID-19	449			**0.004**			**0.010**
No		34.90	37.33		34.33	41.62	
Don't know		25.50	37.33		23.76	35.17	
Yes		39.60	25.33		41.90	23.20	
Barriers for receiving COVID-19 vaccine	448						
Availability		2.70	3.33	0.72	3.39	1.75	0.11
Distance to vaccination point		6.08	5.67	0.86	2.88	1.62	0.21
Cost		0.00	0.00		0.00	0.00	
Not a priority group		6.08	6.67	0.81	2.52	7.11	0.27
Lack of information about how/where to get it		20.95	22.33	0.74	19.50	19.06	0.90
Too stressful		35.14	37.33	0.65	43.82	50.20	0.091
Staff attitude		3.38	3.33	0.98	3.55	3.39	0.95
Not socially acceptable		11.49	36.33	**< 0.001**	11.07	30.41	0.078
None		10.81	11.00	0.95	19.09	14.42	0.39
Would get/have gotten the vaccine if employer recommended	449			**< 0.001**			0.092
No		19.46	43.33		22.43	40.43	
Unsure		6.71	19.33		4.12	21.32	
Yes		73.83	37.33		73.44	38.25	

Values in bold indicate statistically significant results at *p* value < 0.05

^a^Chi-squared test with Rao & Scott's second-order correction

Sources of information on COVID-19 vaccines varied among vaccinated and unvaccinated respondents. Social media was the most common source of information overall (61.64% among the vaccinated and 61.33% among the unvaccinated). Among the respondents receiving information from social media, nearly all used Facebook and around half referenced Instagram. However when asked about information sources on the vaccine, media types including social media, television, radio and newspapers were not associated with vaccination status in the weighted analysis (*p* value > 0.05). There was no difference in social media use among vaccinated and unvaccinated individuals or in the use of specific platforms.

Other common sources of vaccine information were healthcare workers (65.31% of the vaccinated vs. 55.83% of the unvaccinated, *p* = 0.21); neighbors, friends, and colleagues (35.40% of the vaccinated vs. 49.73% of the unvaccinated, *p* < 0.01); and family (30.26% of the vaccinated vs. 37.18% of the unvaccinated, *p* = 0.12). Those who were vaccinated were more likely to report commonly receiving information on the vaccine from newspapers, religious leaders, civil society organizations, and to trust the information received from healthcare providers (OR *p* values < 0.05). The unvaccinated were more likely to receive information from mass events, family, and neighbors, friends, and colleagues (OR *p* values < 0.05).

Multivariable regression provided further evidence for demographic characteristics as independent determinants of vaccination status (Table 9). In the adjusted model, vaccination was around twice as likely among males (aOR 1.88, *p* = 0.006, 95% CI 1.20–2.95), older compared to younger adults (aOR 1.92, *p* < 0.001, 95% CI 1.73–2.13), and the university educated compared to those with primary or no education (aOR 2.19, 95% CI 1.51–3.17). The vaccinated were less likely to report having insufficient information on the vaccine (aOR 0.62, *p* < 0.001, 95% CI 0.48–0.80) as well as more likely to consider the vaccines safe (aOR 13.8, 95% CI 10.1–18.8) or somewhat safe (aOR 5.41, 95% CI 3.69–7.94). However perception of personal risk of developing severe symptoms of COVID-19 was not independently significantly associated with vaccination status (aOR 0.86, 95% CI 0.59–1.24), nor were sources of information, after controlling for demographic characteristics and other risk factors.

### Vaccine hesitancy and risk factors

Vaccine hesitancy was 34.08% (95% CI 28.14–40.56) in the pooled population of vaccinated and unvaccinated individuals,^2^ and 67.24% (95% CI 49.04–81.41) among the unvaccinated sub-group (n = 449).^3^ Table 4 presents the unweighted and weighted distribution of the characteristics of the population by vaccine hesitancy status. Among the overall population, males were less likely to report hesitancy (*p* = 0.006). Other demographic characteristics were not significantly associated with vaccine hesitancy (*p* > 0.05).

Table 6 presents the unweighted and weighted distribution of the risk factors for vaccine hesitancy and Table 8 provides the unadjusted Odds Ratios. The main factors the vaccine hesitant reported as barriers to getting the vaccine were stress associated with the vaccine (51.03%), lack of social acceptance (31.03%), and lack of information on the vaccines (20.45%). However, these barriers were not specifically associated with vaccine hesitancy in unadjusted analysis (*p* > 0.05 for ORs comparing hesitant and non-hesitant individuals), suggesting they were non-specific constraints to vaccine uptake although we were underpowered for this analysis.

Common sources of vaccine information among the hesitant population were social media (58.18%); HCWs (53.29%); friends, neighbors and colleagues (52.52%); and family (41.55%). Notably, those classified as hesitant were more likely to report family (OR 1.68, *p* < 0.001, 95% CI 1.29–2.17); friends, neighbors and colleagues (OR 1.83, *p* = 0.006, 95% CI 1.19–2.82); radio (OR 1.48, *p* = 0.017, 95% CI 1.07–2.03); and mass events (OR 2.28, *p* = 0.023, 95% CI 1.12–4.62) as important sources of information, and less likely to mention HCWs (OR 0.64, *p* = 0.004, 95% CI 0.47–0.86); religious leaders (OR 0.51, *p* = 0.025, 95% CI 0.28–0.92); and civil society organization (OR 0.41, *p* < 0.001, 95% CI 0.24–0.69). They were also more likely to trust family (OR 1.75, *p* < 0.001, 95% CI 1.28–2.40); neighbors, friends and colleagues (OR 3.05, *p* = 0.006, 95% CI 1.37–6.77); and radio (OR 2.87, *p* < 0.001, 95% CI 1.99–4.14) for information on COVID-19 vaccines. They were less likely to trust HCWs (OR 0.54, *p* < 0.001, 95% CI 0.46–0.65); local leaders (OR 0.02, *p* < 0.001, 95% CI 0.00–0.18) and civil society organizations (OR 0.43, *p* < 0.001, 95% CI 0.34–0.56) for accurate vaccine information.

Perceptions of vaccine safety and effectiveness also appear to impact vaccine hesitancy. Those classified as hesitant were far less likely to consider the vaccines safe (2.33% vs. 30.34%, OR 0.03, 95% CI 0.01–0.16). Around 80% of both hesitant and non-hesitant groups believed they were at risk of getting COVID-19; however the hesitant were less likely to consider themselves at risk of severe disease or hospitalization if they contract COVID-19 (OR 0.69, 95% CI 0.50–0.97).

In multivariable regression, the adjusted models showed demographic factors as well as certain information sources and risk perceptions were independently associated with vaccine hesitancy (Table 10). The vaccine hesitant were less likely to be male (aOR 0.58, *p* = 0.004, 95% CI 0.40–0.84), older compared to younger adults (aOR 0.47, *p* = 0.005, 95% CI 0.28–0.80), and university educated compared to primary or no education (aOR 0.32, 95% CI 0.25–0.42). The vaccine hesitant were more likely to mention family as a common source of information on the vaccines (aOR 1.29, *p* = 0.051, 95% CI 1.00–1.67), and less likely to trust HCWs for vaccine information (aOR 0.58, *p* < 0.001, 95% CI 0.49–0.68). Perception of vaccine safety and serious personal risk from COVID-19 are also independently related to vaccine hesitancy. Those who consider the vaccine safe had 97% lower odds of being hesitant (aOR 0.03, 95% CI 0.01–0.15), while those who believed in the possibility of serious illness from COVID-19 had 38% lower odds of being hesitant (aOR 0.62, 95% CI 0.37–1.04).

### Vaccine hesitancy among the non-vaccinated

In sub-analysis, risk factors for vaccine hesitancy were considered for the population of unvaccinated individuals (n = 449) (Table 11). Vaccination outreach efforts will need to specifically target the currently unvaccinated population who are hesitant (~ 67% of this sub-group). In this population, there was not enough evidence that sex, age, or educational background were associated with hesitancy (*p* value > 0.05). There was little variation in the information requested and information sources accessed among hesitant and non-hesitant individuals. The most common sources of information among those classified as hesitant were social media (58.32%), HCWs (53.21%), neighbors, friends and colleagues (52.29%), and family (43.28%).

Perception of vaccine safety and concern about risks of side effects were marginally not significant in the weighted analysis. However the study was not powered to investigate risk factors for vaccine hesitancy among the unvaccinated sub-group. These are likely to be true risk factors despite being underpowered, given the effect size of the estimate and significance in the primary analysis that used the pooled population of vaccinated and unvaccinated individuals. Perception that the virus posed severe individual health risks did vary with hesitancy status. 23.20% of hesitant individuals versus 41.90% of non-hesitant individuals believed they were at risk of serious disease or hospitalization if they contracted COVID-19 (*p* = 0.010).

### Vaccination and vaccine hesitancy among healthcare workers

Table 12 presents the descriptive results for vaccine uptake and risk factors for hesitancy among HCWs. 89.35% (n = 151) of the HCWs surveyed were vaccinated and 10.65% (n = 18) were unvaccinated. Among the non-vaccinated HCWs, 50% (n = 9) were classified as hesitant. Given the small sample of non-vaccinated HCWs, few inferences can be made. However the explanations for hesitancy appear to mirror that of the population. HCWs who were hesitant were less likely to consider the vaccines safe (89% did not consider them safe compared to 11% of non-hesitant HCWs) and were more likely to express concerns about side effects (89% compared to 26% of non-hesitant HCWs).

**Table 12:** Factors associated with vaccine hesitancy, HCWs only

Variable	N	Vaccine hesitant		
		No, N = 160^a^	Yes, N = 9^a^	*p* value^b^
Have enough information about COVID-19 vaccine	169			0.73
Yes		101 (63)	5 (56)	
No or don't know		59 (37)	4 (44)	
Information needed	169			
Eligibility criteria		35 (22)	3 (33)	0.42
Timeline for vaccine roll-out		12 (7.5)	1 (11)	0.52
When it's my turn to get the vaccine		13 (8.1)	1 (11)	0.55
Doses needed		31 (19)	2 (22)	0.69
Risks and side effects		106 (66)	9 (100)	0.059
Effectiveness of the vaccines		106 (66)	7 (78)	0.72
How to register for the vaccine		7 (4.4)	1 (11)	0.36
Common source of information about health and vaccines	169			
Health care providers		132 (82)	6 (67)	0.37
Community health care workers		45 (28)	3 (33)	0.71
Radio		2 (1.2)	0 (0)	> 0.99
Television		14 (8.8)	1 (11)	0.58
Newspapers		2 (1.2)	1 (11)	0.15
Mass events		1 (0.6)	0 (0)	> 0.99
Family		3 (1.9)	0 (0)	> 0.99
Neighbors, friends, colleagues		4 (2.5)	1 (11)	0.24
Local leaders		1 (0.6)	0 (0)	> 0.99
Religious leaders		2 (1.2)	0 (0)	> 0.99
Social media		51 (32)	5 (56)	0.16
Organizations		59 (37)	2 (22)	0.49
Trusted source of information	169			
Health care providers		118 (74)	5 (56)	0.26
Community health care workers		30 (19)	2 (22)	0.68
Local leaders		0 (0)	0 (0)	
Religious leaders		0 (0)	0 (0)	
Family members		0 (0)	1 (11)	0.053
Neighbors, friends, colleagues		2 (1.2)	1 (11)	0.15
Organizations		55 (34)	1 (11)	0.27
Media		10 (6.2)	2 (22)	0.13
Radio		2 (1.2)	0 (0)	> 0.99
Television		5 (3.1)	0 (0)	> 0.99
Other		15 (9.4)	1 (11)	0.60
Trust health providers and CHWs to provide with accurate information about the COVID-19 vaccine	169			**< 0.001**
No or don't know		9 (5.6)	5 (56)	
Yes		106 (66)	1 (11)	
Somewhat		45 (28)	3 (33)	
Follow social media platforms to get info about vaccine	169			> 0.99
No or don't know		42 (26)	2 (22)	
Yes		118 (74)	7 (78)	
Social media platforms used to get info about vaccine	125			
Facebook		114 (97)	7 (100)	> 0.99
Twitter		18 (15)	1 (14)	> 0.99
Instagram		38 (32)	2 (29)	> 0.99
YouTube		29 (25)	3 (43)	0.37
Shares information related to vaccine on social media network	166	53 (34)	1 (11)	0.27
How do you know info about vaccine from social media is true	168			**0.034**
I do not verify		17 (11)	4 (44)	
Other		11 (6.9)	0 (0)	
Verify from reputable website or health care provider		131 (82)	5 (56)	
Do leaders (religious, political, teachers, health care workers) in your community support the COVID-19 vaccines?	107			> 0.99
No or don't know		20 (19)	0 (0)	
Yes		84 (81)	3 (100)	
Most people you know interested in getting the COVID-19 vaccine	169			0.072
No or don't know		35 (22)	5 (56)	
Yes		87 (54)	3 (33)	
Somewhat		38 (24)	1 (11)	
How many people in your community are concerned about COVID-19 in the community	135			0.80
Few or some people, less than half		50 (39)	2 (29)	
Most people		37 (29)	3 (43)	
Some people—more than half		41 (32)	2 (29)	
COVID-19 vaccines will or are being rolled out equitably in community	169			0.058
No or don't know		40 (25)	5 (56)	
Yes		120 (75)	4 (44)	
Knows somebody that had a serious negative reaction to a vaccine that makes them reluctant to get COVID-19 vaccine	167			**0.049**
Yes		24 (15)	4 (44)	
Somewhat		20 (13)	1 (11)	
No or don't know		114 (72)	4 (44)	
Consider COVID-19 vaccines safe	169			**< 0.001**
No or don't know		18 (11)	8 (89)	
Yes		74 (46)	0 (0)	
Somewhat		68 (42)	1 (11)	
Concerned about risks or side effects with COVID-19 vaccines	169			**< 0.001**
No or don't know		75 (47)	1 (11)	
Yes		42 (26)	8 (89)	
Somewhat		43 (27)	0 (0)	
Types of risks or side effects concerned about	92			
Fever		44 (52)	4 (50)	> 0.99
Body aches		55 (65)	4 (50)	0.45
Infertility		16 (19)	3 (38)	0.35
Physical disability		10 (12)	2 (25)	0.28
Death		10 (12)	6 (75)	**< 0.001**
Other		18 (21)	4 (50)	0.090
Type of COVID vaccine preferred	169			0.35
Other		0 (0)	0 (0)	
No preference		16 (10)	2 (22)	
Pfizer		93 (58)	3 (33)	
Sputnik		40 (25)	4 (44)	
Moderna		8 (5.0)	0 (0)	
Unsure		3 (1.9)	0 (0)	
Sinopharm		0 (0)	0 (0)	
Believe there are other (better) ways to prevent COVID-19 instead of vaccine	169			0.48
No or don't know		79 (49)	3 (33)	
Yes		52 (32)	5 (56)	
Somewhat		29 (18)	1 (11)	
Better ways to prevent COVID-19 instead of vaccine	57			
Social distance		45 (87)	3 (60)	0.17
Handwashing		45 (87)	4 (80)	0.54
Infection prevention and control		22 (42)	3 (60)	0.64
Ventilation		18 (35)	2 (40)	> 0.99
Wearing face masks		42 (81)	4 (80)	> 0.99
Know any person with a serious disease/disability that happened because they were NOT vaccinated	167			0.27
Yes		52 (33)	1 (11)	
No or don't know		106 (67)	8 (89)	
Think it is better to get COVID-19 and develop natural immunity than to get the vaccine	169			0.12
No		87 (54)	2 (22)	
Don't know		22 (14)	1 (11)	
Yes		38 (24)	5 (56)	
Somewhat		13 (8.1)	1 (11)	
Remember past events that would discourage them from getting COVID-19 vaccine	18			0.58
Yes		1 (11)	3 (33)	
No or don't know		8 (89)	6 (67)	
Think you are at risk to get COVID-19	168			0.63
No		5 (3.1)	0 (0)	
Don't know		11 (6.9)	1 (11)	
Yes		143 (90)	8 (89)	
Think you can get seriously ill, hospitalize or die if you get COVID-19	169			> 0.99
No		67 (42)	4 (44)	
Don't know		53 (33)	3 (33)	
Yes		40 (25)	2 (22)	
Barriers for receiving COVID-19 vaccine	18			
Availability		0 (0)	0 (0)	
Distance to vaccination point		1 (11)	1 (11)	> 0.99
Cost		0 (0)	0 (0)	
Not a priority group		0 (0)	1 (11)	> 0.99
Lack of information about how/where to get it		1 (11)	2 (22)	> 0.99
Too stressful		1 (11)	0 (0)	> 0.99
Staff attitude		0 (0)	0 (0)	
Not socially acceptable		0 (0)	1 (11)	> 0.99
None		1 (11)	0 (0)	> 0.99
Would get/have gotten the vaccine if employer recommended	18			0.35
No		3 (33)	5 (56)	
Unsure		0 (0)	1 (11)	
Yes		6 (67)	3 (33)	

Values in bold indicate statistically significant results at *p* value < 0.05

^a^Frequency (%)

^b^Fisher's exact test

## Discussion

The beginning of the global roll-out of COVID-19 vaccines in 2021 was met by calls to ensure equitable access for less developed countries, refugees, and internally displaced populations [25]. Disparities nonetheless became apparent within the first year [26]. According to the United Nations, as of November 2021 only 4% of COVID-19 vaccine doses had been administered in the 30 countries prioritized for multilateral humanitarian assistance [27]. The evidence base on COVID-19 vaccines from these types of settings also remains limited. Most reports publicly available consist of basic vaccine administration metrics, or otherwise provide descriptive or semi-qualitative results such as a recent study in Yemen [28].

This is one of the first analytical studies of COVID-19 vaccine coverage and hesitancy from a humanitarian setting. We estimated vaccine coverage in Gaza to be 49.09% (95% CI 43.10–55.08) of the adult population. This estimate is comparable, although slightly higher, than the 44% figure reported by the WHO in its February 2022 situation report [13]. The difference could be largely attributed to the methods, whereby the WHO estimates the percentage of the population vaccinated by comparing routine vaccine administration data to population statistics. Our survey was also restricted to adults 18 years and older, while the WHO data encompasses adolescents 12 years and older who were not eligible in the early phases of vaccine roll-out. Although there is no data on parental hesitancy in Gaza, adolescents may also be less likely to receive the COVID-19 vaccine compared to adults due to parental reluctance that has been observed globally and varies across contexts [29, 30].

The study’s large sample size also enabled reasonable sub-group estimates of vaccination coverage. This is useful because routine data on COVID-19 vaccine distribution, especially from lower income countries, has generally not been disaggregated, which prevents analysis of within-country inequities [31]. In Gaza, we found moderate to large differences in vaccine coverage for each demographic characteristic assessed. Women were less likely to be vaccinated than men, with 41.45% of females vaccinated compared to 54.87% of males. Routine data from vaccine administration in the oPt corroborates this trend, as females comprised just 44% of people who received a vaccine dose as of February 2022 [13]. People with lower levels of education were also less likely to be vaccinated—37.30% of those with primary or no education compared to 43.12% of those with secondary or college education and 55.75% of those with university education. These findings suggest certain systemic inequities observed in global vaccination programs may be occurring with COVID-19 vaccination, at least in Gaza, and warrants attention in other settings. A recent meta-analysis on vaccine equity in low and middle-income countries found lower likelihood of vaccination among females, households with lower education, and poorer households [32].

In terms of hesitancy, we estimated the vaccine hesitant consist of 34.08% (95% CI 28.14–40.56) of the adult population of Gaza, which is similar to the estimate from the web-based survey (37%) conducted in Gaza in October 2020 [11]. Temporal data from vaccine hesitancy studies have generally shown levels of hesitancy reported within countries to decrease over time as vaccines are rolled out and the populations gain first-hand experience [5]. While limited comparison can be drawn to the web survey, our similar figures suggest COVID-19 vaccine confidence in Gaza has been persistently low following the initial vaccine roll-out. Follow-up surveys are warranted to monitor the coverage and effectiveness of vaccine promotion efforts.

Among the non-vaccinated, 67.24% were classified vaccine hesitant, suggesting they will be difficult to reach through improved vaccine supply alone. Characteristics of the vaccine hesitant in Gaza mirror global findings, with less education as well as lower perception of serious risk from COVID-19 linked to vaccine hesitancy [33, 34]. Among the non-vaccinated group, those classified as hesitant were less likely to consider the vaccine safe and less likely to believe they were at risk of severe disease—suggesting their perceived risk of vaccination outweighed perceived risk of infection. They are also more likely to rely on family and friends for vaccine information—a finding that mirrors the global scoping review conducted by Biwas et al. [5]. Conversely, perception of personal benefit and effectiveness of the vaccines, as well as lower concern about side effects, appears to positively influence vaccination.

Globally, there remains uncertainty about the associations between demographic characteristics and vaccine hesitancy. Research is conflicting on relative levels of vaccine hesitancy among different demographic groups and appears to be context specific. Our study was not powered to specifically investigate risk factors among non-vaccinated and vaccine hesitant sub-groups. Recent studies from the Middle East have reported females more likely to be vaccine hesitant, which our study similarly found in the primary analysis [7, 8, 34]. However, vaccination rates were also notably lower among women compared to men, and sex was not associated with vaccine hesitancy among the non-vaccinated sub-group alone. The difference in the sub-analysis suggests unvaccinated women may encounter unique barriers to accessing vaccines compared to women who are vaccinated. Additional research is warranted to assess potential explanatory factors such as economic empowerment, social freedoms, and access to information. Age was also not associated with vaccine hesitancy among the non-vaccinated in Gaza, whereas other studies have found younger groups to be more vaccine hesitant or less motivated to pursue vaccination [33]. As our study was not powered for sub-group analyses, though, it is possible that larger surveys would identify further variations.

We found high COVID-19 vaccination coverage (89%) among HCWs, similar to global reports [35, 36]. Despite the smaller number of HCWs interviewed for our study and use of purposive sampling, the high level of uptake is likely a valid representation given the vaccine mandates for this group. From June 2021, the de facto government in Gaza Strip required staff of the Ministry of Health (MoH) and other agencies to be vaccinated against COVID-19. Therefore among this group, vaccination is not necessarily a valid measure of vaccine hesitancy; a non-negligible proportion of HCWs expressed doubts about certain aspects of the vaccine. More than 15% of HCWs stated they do not consider the vaccine safe or do not know, while one-quarter of HCW respondents reported concern about side effects. Among non-vaccinated HCWs, perception of insufficient vaccine safety and efficacy appear to be major determinants of hesitancy. Recent articles by El Kibbi et al. and Heyerdahl et al. raised concerns on “unspoken vaccine hesitancy” among HCWs in the Middle East, which our own study’s findings modestly support as a subject worth monitoring [37, 38].

A major strength of our study is its broad scope which includes vaccine coverage estimates, vaccine hesitancy, comparisons among sub-groups of the populations, and risk factors associated with both vaccination and vaccine hesitancy. The findings can be used to develop more persuasive and effective information campaigns, and to proactively address the concerns of vaccine hesitant sub-groups through the information channels they most commonly use and trust. Subsequent research in Gaza could examine changes in vaccine hesitancy over time and evaluate the effectiveness of outreach campaigns and social messaging based on the emerging evidence of risk factors for vaccine hesitancy.

## Limitations

The main limitation of the study is the non-standard approach to cluster sampling, whereby the clusters were selected with probability proportional to size of governorates rather than size of clusters, and the number of geo-localities selected per strata was limited to two in each stratum. We partly account for sampling bias in analysis through the sampling weights. The reliance on convenience sampling of primary survey respondents within households rather than purely random sampling of adult household members has potential for selection bias. Characteristics of people who were available and willing to participate in the survey may have differed from a random sample of eligible household members and the general population. In areas where socio-cultural norms reflect greater male dominance, men may have been more likely to answer the door or respond to the survey. The survey also excluded people who were working or moving outside the house at the time of the survey. While the direction of potential bias related to the sampling design is unclear, prevalence values and model results should be interpreted with caution.

Similarly, we collected hesitancy information from healthcare workers to investigate coverage and hesitancy among this at-risk group of frontline responders. Operational constraints in Gaza necessitated a non-random selection of health facilities that were accessible to the researchers. Due to the sampling design, the findings of the HCW survey should not be considered representative of the health workforce of Gaza. While the facilities included in our study reasonably reflect the different types of primary and secondary health services available in the territory, they are likely to differ from a random sample in terms of their financial means, workforce training, and connectedness to humanitarian aid systems. The respondents within each facility were also a self-selected sample of eligible HCWs, who may differ systematically from staff who chose not to take the survey. For example, stigma regarding vaccine hesitancy among HCWs may have driven a lower survey response rate among staff who were hesitant.

Another limitation was the use of a standard global tool for assessment of vaccine hesitancy and confidence. As such, not all reasons for hesitancy were factored into the survey and questions were not customized to the context of the Gaza Strip. We did not inquire about previous SARS-CoV-2 infection and history of COVID-19 disease, comorbidities, movement restrictions in Gaza, the recent conflict, and other individual and local factors that could influence vaccine hesitancy. The standard vaccine hesitancy tool was also not customized for the interviews with healthcare workers, so certain factors more relevant to this population—such as clinical and epidemiological knowledge, patient care experience, and employer mandates—that correlate with vaccine acceptance were not assessed here.

## Conclusion

The recurrent and rapid emergence of COVID-19 variants reinforces the urgency to accelerate vaccination efforts in under-resourced and conflict-prone settings like the Gaza Strip. Scientists have linked recent variants such as Delta and Omicron to low and inequitable vaccination coverage globally, indicating the need for both supply- and demand-oriented strategies to combat the pandemic [39, 40]. Our study complements previous research on the latter through an investigation of vaccination coverage and vaccine hesitancy in the difficult public health setting of the Gaza Strip. It is one of the first population-representative studies of these topics in the Middle East and is also the first carried out in Gaza since the vaccine roll-out.

Just half of adults in Gaza had received at least one dose of a vaccine by October 2021. Disparities in vaccination coverage were apparent across the territory’s social and demographic groups; vaccination campaigns should subsequently target those who are less likely to be vaccinated, including women and those with less education. HCWs are a widely trusted information channel on COVID-19 vaccines among the vaccinated and unvaccinated, although less trusted among those who are hesitant. The latter reported that they accessed information from family, friends, neighbors, and colleagues at higher levels than the vaccinated and non-hesitant. This suggests the importance of disseminating accurate messages through community-based channels to permeate social networks of the unvaccinated and to address misinformation from these sources. Health promotion strategies to encourage vaccination should emphasize vaccine effectiveness and minimal risk of serious side effects, both of which were major concerns voiced by the unvaccinated and vaccine hesitant. The optimal approach is likely to be a combination—mobilizing people who are already well-trusted by vaccine hesitant individuals and training these potential influencers on specific topics linked to vaccine hesitancy.

## Supplementary Information

**Additional file 1.** Data collection instrument (original English and Arabic translation).

**Additional file 2.** Flow chart of multi-stage sampling approach used for community survey.

**Additional file 3.** Population of governorates and geolocalities in Gaza as used to calculate sample size of clusters. Administrative data provided by PCBS.

**Additional file 4.** Finite population correction (FPC) and sampling weights per cluster.

## Data Availability

The datasets used and analysed during the current study are available from the corresponding author’s institution on reasonable request.

## Abbreviations

CHW: Community Health Worker
HCW: Healthcare Workers
OR: Odds ratio
PCBS: Palestinian Central Bureau of Statistics
PWD: Persons with Disabilities
IMC: International Medical Corps
MoH: Ministry of Health
oPt: occupied Palestinian Territory
WHO: World Health Organization

## Footnotes

1 Geo-locations are comparable to administrative level 3, below the governorate level.

2 Respondents who answered “No” or “Not sure” to the question, “If you could get the COVID-19 vaccine this week, would you get it? were classified “hesitant”. Those answering “Yes” as well as currently vaccinated people were classified “non-hesitant.”

3 Currently unvaccinated respondents who answered “No” or “Not sure” to the question, “If you could get the COVID-19 vaccine this week, would you get it? “were classified “hesitant”, while those answering “Yes” were classified “non-hesitant.” Currently vaccinated individuals were excluded.
