# Supplementary material for: COVID-19 vaccination in the Gaza Strip: a cross-sectional study of vaccine coverage, hesitancy, and associated risk factors among community members and healthcare workers
Source: Confl Health. 2022 Sep 9;16:48. doi: 10.1186/s13031-022-00477-7 (PMC9461392; doi:10.1186/s13031-022-00477-7)
Supplement: Supplementary file 1 — Additional file 1. Data collection instrument (original English and Arabic translation). [file 13031_2022_477_MOESM1_ESM.docx]

**S1.a. Vaccine Hesitancy Assessment Quantitative Survey Tool**

| **Enumerator Number:** |  |
| --- | --- |
| **Location^[[1]](#footnote-1)^:** |  |
| **Cluster number^[[2]](#footnote-2)^:** |  |
| **HH number:** |  |
| **Date:** |  |
| **Time of visit:** |  |
| **Project name** |  |
| **READ:** Hello I am working on a study for IMC XXX^[[3]](#footnote-3)^. The purpose of the study is to collect information to better understand perceptions of community members and health care workers on COVID-19 vaccines, and to assess interest in COVID-19 vaccination and reasons for hesitancy and concerns. I would like to ask you some questions about your perception of the COVID-19 vaccines. Your answers are confidential and cannot be linked back to you. Your participation is completely voluntary and you may decline to answer any specific question or completely refuse to participate. The interview should take about 30 minutes of your time and you will not be contacted in the future. We would greatly appreciate your help in responding to these questions. | |
| **Are you willing to be interviewed at this time?** | ☐ 1. Yes  ☐ 2. No |

| **Background Questions^[[4]](#footnote-4)^** | |
| --- | --- |
| A1. Do you usually reside in this household? | ☐ 1. Yes  ☐ 2. No (ask to speak with a resident of the household) |
| A2. What is your relationship to Head of Household? | ☐ 1. Is the Head of household  ☐ 2. Spouse  ☐ 3. Child  ☐ 4. Grand child  ☐ 5. Parent  ☐ 6. Sibling  ☐ 0. Other Relative __________________________  ☐ 8. Not related  ☐ 98. Don’t Know |
| A3. Respondent Gender | 1. Female  2. Male  0. Other _________________________________  4. Prefer not to say |
| A4. Respondent Age  Note: If the respondent is <18 and not the head of household, ask to speak with the primary caregiver or head of household. | 1. 15-17 (child headed household)  2. 18-29  3. 30-39  ☐ 4. 40-49  ☐ 5. 50+ |
| A5. What is your current marital status? | ☐ 1. Married  ☐ 2. Living together  ☐ 3. Separated/Divorced  ☐ 4. Widowed  ☐ 5. Single  ☐ 98. Don’t Know |
| A6. How many people live in the household including children? | ☐ Number of children <2 years __________  ☐ Number of children 2- <5 years __________  ☐ Number of children 5-17 years ____________  ☐ Number of adults 18+ years _______________ |
| A7. Do you have any of these members living in your household? | ☐ 1 Pregnant/lactating women  ☐ 2 Persons with disabilities  ☐ 3 Persons with chronic illnesses  ☐ 4 Elderly |
| A8. Have you ever attended school? | ☐ 1. Yes  ☐ 2. No  ☐ 98. Don’t Know |
| A9. What is your highest educational grade/level that you have completed? | ☐ 1. None/Never attended school  ☐ 2. Adult Literacy  ☐ 3. Primary  ☐ 4. Secondary  ☐ 5. College/Vocational  ☐ 6. University |
| A10. What is the primary language spoken in your household? |  |
| A11. Have you already received COVID-19 vaccine?  (if yes, skip E1-E3, G1 and G2) | ☐ 1. Yes  ☐ 2. No  ☐ 98. Don’t Know |
| A11a. If yes, when? | ☐ 1. A week ago or less  ☐ 2. Two weeks ago  ☐ 3. 3-4 weeks ago  ☐ 4. More than a month ago |
| A11b. If yes, what vaccine? | ☐ 1 Pfizer/BioNtech  ☐ 2 Moderna  ☐ 3 Oxford-AstraZeneca  ☐ 4 Johnson and Johnson  ☐ 5 Novavax  ☐ 6 Sinovac  ☐ 7 Sinopharm  ☐ 8 Sputnik V  ☐ 9 CanSinoBIO  ☐ 10 Unsure  ☐ 0 Other (Please specify: _____________) |
| A12. How many doses have you received? | ☐ 1. One dose  ☐ 2. Two doses |
| **Individual Knowledge** |  |
| B1. Do you have enough information about the vaccine in your country /community? | ☐ 1. Yes  ☐ 2. No  ☐ 98. Don’t Know |
| B2. What information would you be interested in/useful for you? | ☐ 1 Eligibility criteria  ☐ 2 Timeline for roll-out  ☐ 3 How will I know when it’s my turn  ☐ 4 How many doses do I need  ☐ 5 Risks and side effects  ☐ 6 Effectiveness of the vaccines  ☐ 7 How to register for the vaccine?  ☐ 0 Other (*Specify: ____________________*) |
| B3. What are the most common sources of information you use to learn about health and vaccines? (select three, starting with most important) | ☐ 1 Health care providers / staff at health clinics  ☐ 2 Community health care workers  ☐ 3 Radio  ☐ 4 Television  ☐ 5 Newspapers  ☐ 6 Mass events  ☐ 7 Family  ☐ 8 Neighbors, friends, colleagues  ☐ 9 Local leaders  ☐ 10 Religious leaders  ☐ 11 Social media *(Specify: ______________)*  ☐ 12 Organizations *(Specify: ______________*)  ☐ 0 Other *(Specify: ______________*) |
| B4. Which source do you trust? | ☐ 1 Health care providers / staff at health clinics  ☐ 2 Community health care workers  ☐ 3 Community leaders (*Specify position:__________)*  ☐ 4 Radio  ☐ 5 Television  ☐ 6 Newspapers  ☐ 7 Mass events  ☐ 8 Local leaders  ☐ 9 Religious leaders  ☐ 10 Social media *(Specify: ______________)*  ☐ 11 Organizations *(Specify: ______________*)  ☐ 0 Other *(Specify: ______________*) |
| B5. Do you follow social media platforms (Facebook, Twitter, Instagram, YouTube) and website to get information about the Coronavirus vaccine? | ☐ 1 Yes  ☐ 2 No  ☐ 98. Don’t Know |
| B6. If yes, which platforms do you use? | ☐ 1 Facebook  ☐ 2 Twitter  ☐ 3 Instagram  ☐ 4 YouTube  ☐ 0 Other (*Specify: ____________________*) |
| B7. Do leaders (religious, political, teachers, health care workers) in your community support the COVID-19 vaccines? | ☐ 1 Yes  ☐ 2 Some do, others don’t  ☐ 3 No  ☐ 98 Don’t know |
| B8. If yes, do you agree with them? | ☐ 1 Yes  ☐ 2 Somewhat  ☐ 3 No  ☐ 98 Don’t know |
| B9. Do you feel that you have enough information about the COVID-19 vaccine? | ☐ 1 Yes  ☐ 2 Somewhat  ☐ 3 No  ☐ 98 Don’t know |
| B10. Are most people you know interested in getting the COVID-19 vaccine? | ☐ 1 Yes  ☐ 2 Somewhat  ☐ 3 No  ☐ 98 Don’t know |
| **Community Perception** | |
| C1. Do you share information related to the COVID-19 vaccine within your social media network? | ☐ 1 Yes  ☐ 2 No  ☐ 3 NA |
| C2. If yes, what type of information?  *(choose all that apply)* | ☐ 1 Availability  ☐ 2 Concerns about side effects  ☐ 3 Information about usefulness  ☐ 4 Discouraging others from taking it  ☐ 5 Encouraging others to take it  ☐ 6 Getting yourself registered  ☐ 0 Other (*Specify: ________________)* |
| C3. When you get information about the COVID-19 vaccine through social media, how do you know if it is true? | ☐ 1 Verify from reputable website  ☐ 2 Verify through health care provider  ☐ 3 I trust information whatever I receive  ☐ 4 Ask sender to provide the source  ☐ 5 I do not verify  ☐ 0 Other *(Specify: ________________)* |
| C4. Approximately how many people in the community do you think are concerned about the spread of COVID-19 in the community? community perception | ☐ 1 Most people  ☐ 2 Some people – more than half  ☐ 3 Some people – less than half  ☐ 4 Few people |
| C5. Do you think the COVID-19 vaccines will or are being rolled out equitably to your specific community? | ☐ 1 Yes  ☐ 2 No  ☐ 98 Don’t know |
| **Convenience** | |
| D1. Would you get/have gotten the vaccine if it was offered at the: (select all that apply): | ☐ 1 Malls  ☐ 2 Places of worship (churches, mosques, etc.)  ☐ 3 Workplace  ☐ 4 None of the above |
| D2. Would you prefer to get/have gotten the vaccine if it was offered at the: (select one) | ☐ 1 Mobile vaccination units  ☐ 2 PHCs  ☐ 3 Hospitals  ☐ 4 No preference |
| D3. What barriers do you face (or foresee) for receiving the COVID-19 vaccine? | ☐ 1 Availability  ☐ 2 Distance to vaccination point  ☐ 3 Cost  ☐ 4 Other responsibilities  ☐ 5 Not a priority group  ☐ 6 Lack of information about how / where to get it  ☐ 7 Staff attitude  ☐ 8 Not socially acceptable  ☐ 9 Too stressful  ☐ 10 None  ☐ 0 Other (specify) |
| **Confidence** | |
| E1. Do you think vaccines will protect you from COVID-19? | ☐ 1 Yes  ☐ 2 No  ☐ 3 Somewhat |
| E2. Would you get/have gotten the vaccine if your employer recommended it? | ☐ 1 Yes  ☐ 2 No  ☐ 3 Unsure |
| E3. If not already taken, would you get the COVID-19 if you saw that your neighbors, community leaders, religious leaders, doctors, celebrities, politicians, etc. had already received the vaccine? | ☐ 1 Yes  ☐ 3 No  ☐ 98 Don’t know |
| E4. Do you remember any events in the past that would discourage you from getting the COVID-19 vaccine? | ☐ 1 Yes  ☐ 2 No |
| E5. Do you consider the COVID-19 vaccines safe? | ☐ 1 Yes  ☐ 2 No |
| E5a. If yes, please describe | ☐ 1 Had a negative experience with a vaccine  ☐ 2 Knew somebody that had a negative experience with a vaccine  ☐ 3 Had a negative experience with healthcare workers / health facilities  ☐ 0 Other *(Please specify: _____________)* |
| E6. Has someone you know ever had a serious negative reaction to a vaccine which makes them you reluctant to get the COVID-19 vaccine? | ☐ 1 Yes  ☐ 2 Somewhat  ☐ 3 No  ☐ 98 Don’t know |
| E7. Do you consider the COVID-19 vaccines safe? | ☐ 1 Yes  ☐ 2 Somewhat  ☐ 3 No  ☐ 98 Don’t know |
| E8. Are you concerned about any risks or side effects with the COVID-19 vaccine? | ☐ 1 Yes  ☐ 2 Somewhat  ☐ 3 No  ☐ 98 Don’t know |
| E8a. If yes or somewhat, what kind of risks or side effects? | ☐ 1 Fever  ☐ 2 Body aches  ☐ 3 Infertility  ☐ 4 Physical disability  ☐ 5 Death  ☐ 0 Other *(Please specify: _____________)* |
| E9. Is there a type of COVID vaccine that you would prefer? | ☐ 1 Pfizer/BioNtech  ☐ 2 Moderna  ☐ 3 Oxford-AstraZeneca  ☐ 4 Johnson and Johnson  ☐ 5 Novavax  ☐ 6 Sinovac  ☐ 7 Sinopharm  ☐ 8 Sputnik V  ☐ 9 CanSinoBIO  ☐ 10 No preference  ☐ 11 Unsure  ☐ 0 Other *(Please specify: _____________)* |
| E10. Do you believe that there are other (better) ways to prevent COVID-19 instead of the vaccine?  (if no or don’t know, skip to E11) | ☐ 1 Yes  ☐ 2 Somewhat  ☐ 3 No  ☐ 98 Don’t know |
| E10a. If yes or somewhat, what are these ways? *(choose all that apply)* | ☐ 1 Social distancing  ☐ 2 Handwashing  ☐ 3 Infection prevention and control  ☐ 4 Ventilation  ☐ 5 Wearing face masks  ☐ 0 Other *(Please specify: _____________)* |
| E11. Do you trust your health providers and CHWs to provide you with accurate information about the COVID-19 vaccine? | ☐ 1 Yes  ☐ 2 No  ☐ 3 Somewhat  ☐ 98 Don’t know |
| E12. Do you think your health system can safely administer the COVID-19 vaccine to the population? | ☐ 1 Yes  ☐ 2 No  ☐ 3 Somewhat  ☐ 98 Don’t know |
| **Complacency** | |
| F1. Do you think you are at risk to get COVID-19? | ☐ 1 Yes  ☐ 2 No  ☐ 3 Somewhat  ☐ 98 Don’t know |
| F2. Do you think you can get seriously ill, hospitalized or die if you get COVID-19? | ☐ 1 Yes  ☐ 2 No  ☐ 3 Somewhat  ☐ 98 Don’t know |
| F3. Do you know of any person with a serious disease/ disability that happened because they were NOT vaccinated? | ☐ 1 Yes  ☐ 2 No  ☐ 98 Don’t know |
| F4. Do you think it is better to get COVID-19 and develop natural immunity than to get the vaccine? | ☐ 1 Yes  ☐ 2 No  ☐ 3 Somewhat  ☐ 98 Don’t know |
| **Intent** | |
| G1. Would you delay vaccinating yourself with a newly introduced/ recommended vaccine? | ☐ 1 Yes  ☐ 2 No  ☐ 98 Don’t know |
| **G2. If the vaccine was available, would you get it?**  **(if gotten the vaccine, skip to the end)** | ☐ 1 Yes  ☐ 2 No  ☐ 98 Unsure |
| G3. Do you have any comments or feedback about COVID-19 vaccine that you would like to share with us? |  |

**Thank you for participating in this survey!**

Enumerator comments:

___________________________________________________________________________________

___________________________________________________________________________________

| 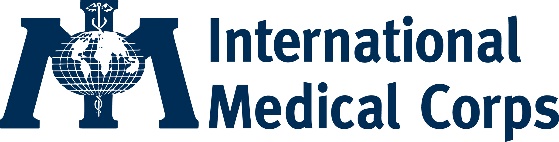 |  |
| --- | --- |

: **S1.a.استبيان حول توجهات الافراد تجاه لقاح كوفيد 19**

|  | رقم العداد (مجري المقابلة) |
| --- | --- |
|  | الموقع |
|  | رقم الكتلة |
|  | رقم الأسرة |
|  | التاريخ |
|  | وقت الزيارة |
|  | اسم المشروع |
| إقرأ: تهدف هذه الدراسية الى فهم توجهات المجتمع و العاملين في القطاع الصحي تجاه لقاحات كوفيد-19 وفهم تردد البعض لأخد اللقاح واسباب هذا التردد. سنقوم بسؤالك بعض الأسئلة عن معتقداتك الخاصة باللقاح - ستكون مشاركتك اختيارية و سرية وبإمكانك عدم الإجابة عن أي سؤال من الأسئلة او رفض المشاركة بشكل تام ولن يتم مراجعتك بهذا الخصوص. تستغرق المقابلة حوالي 30 دقيقة من وقتك ولن يتم الاتصال بك في المستقبل. سنكون ممتنين لمساعدتكم في الرد على هذه الأسئلة | |
| نعم  لا | هل ترغب باالمشاركة ومتابعة الاستبيان؟ |

|  | **أسئلة أساسية** |  | |
| --- | --- | --- | --- |
| A1 | هل تقيم عادة في هذا المنزل؟ | 1. نعم 2. لا (اطلب التحدث مع أحد سكان الأسرة) | |
| A2 | ما علاقتك برب الأسرة؟ | 1. رب الأسرة 2. زوج\زوجة 3. طفل 4. طفل حفيد 5. الأبوين 6. الأخ أو اخت 7. قريب أخر------------- 8. ليس قريب 9. لا اعرف | |
| A3 | جنس المستجيب | 1. ذكر 2. انثي 3. أخرى 4. يفضل عدم الإجابة | |
| A4 | عمر المستجيب  ملاحظة: إذا كان المستجيب أقل من 18 عامًا وليس رب الأسرة ، فاطلب التحدث مع مقدم الرعاية الأساسي أو رب الأسرة | 1. 15- 17 (الطفل رب الأسرة) 2. 18- 29 3. 30- 39 4. 40- 49 5. +50 | |
| A5 | ما هو الوضع العائلي الحالي؟ | 1. متزوج 2. العيش مع بعض 3. مطلق/ة - منفصل/ة 4. ارمل/ة 5. اعزب /عزباء   98..لا اعرف | |
| A6 | كم عدد الأشخاص الذين يعيشون في المنزل بما في ذلك الأطفال؟ | 1. عدد الأطفال أقل من سنتيين 2. عدد الأطفال من 2 – أقل من 5 سنوات 3. عدد الأطفال من 5 – 17 سنة 4. عدد البالغين 18 سنة فأكثر | |
| A7 | هل لديك أي من هؤلاء الأفراد يعيشون في منزلك؟ | 1. سيدة حامل /مرضعة 2. أشخاص معاقين 3. أشخاص يعانون من أمراض مزمنة 4. كبار السن | |
| A8 | هل سبق لك أن التحقت بالمدرسة؟ | 1. نعم 2. لا   98. لا أعلم | |
| A9 | ما هي أعلى درجة / مستوى تعليمي أكملته؟ | 1. لاشئ لم بلتحق بالمدرسة 2. محو أمية الكبار 3. الإبتائية 4. ثانوية عامة 5. الكلية المهنية 6. الجامعة | |
| A10 | ما هي اللغة الأساسية المستخدمة في منزلك؟ |  | |
| A11 | هل تلقيت بالفعل لقاح COVID-19؟  (if yes, skip E1-E3, G1 and G2) | 1. نعم 2. لا   98. لا أعلم | |
| A11a | اذا كان نعم، متى؟ | 1. منذ اسبوع أو أقل 2. منذ أسبوعين 3. منذ 3- 4 أسابيع 4. منذ أكثر من شهر | |
| A11b | اذا كان نعم، ما هو اللقاح؟ | 1. فايزر\بيونتك 2. مودرنا 3. أسترا زينيكا اكسفورد 4. جونسون جونسون 5. نوفا فاكس 6. سينوفاك 7. سبنوفارم 8. سبوتنك 9. كان سينو بيو 10. غير متأكد 11. أخرى (حدد--------------------) | |
| A12 | كم جرعة تلقيت؟ | 1. واحدة 2. اثنتان | |
|  | **أسئلة للمعرفة** |  | |
| B1 | هل لديك معلومات كافية عن اللقاح في بلدك\مجتمعك؟ | 1. نعم 2. لا 3. لا أعرف | |
| B2 | ما هي المعلومات التي ترغب بها ـأو مفيدة لك؟ | 1. معايير الأهلية 2. الجدول الزمني لبدء التنفيذ 3. كيف سأعرف عندما يحين دوري 4. كم عدد الجرعات التي أحتاجها 5. مخاطر وأعراض جابية 6. فاعلية اللقاحات 7. كيف يتم التسجيل للحصول على اللقاح   98. أخرى (حدد------------------------------) | |
| B3 | ما هي أكثر مصادر المعلومات شيوعًا التي تستخدمها للتعرف على الصحة واللقاحات؟ (حدد ثلاثة ، بدءًا من الأكثر أهمية؟ | 1. مزودي الرعاية الصحية والعاملين في المنشآت الصحية 2. العاملين الصحيين المجنمعيين 3. الراديو 4. التلفزيون 5. الصحف 6. المناسبات العامة 7. العائلة 8. الجيران والأقارب والاصدقاء 9. قادة محليين 10. الشيوخ والأئمة 11. وسائل التواصل الاجتماعي -حدد في الفراغ ادناه------------------------------ 12. المؤسسات (حدد ---------------) 13. أخرى (حدد----------------------) | |
| B4 | ما هو المصدر تثق به؟ | 1. مزودي الرعاية الصحية والعاملين في المنشآت الصحية 2. العاملين الصحيين المجنمعيين 3. قادة المجتمع (حدد الوظيفة-------------) 4. الراديو 5. التلفزيون 6. الصحف 7. المناسبات العامة 8. قادة محليين 9. الشيوخ والأئمة 10. وسائل التواصل الاجتماعي -حدد ---------- 11. المؤسسات (حدد ---------------) 12. أخرى (حدد----------------------) | |
| B5 | هل تتابع منصات التواصل الاجتماعي (فيسبوك ، تويتر ، إنستجرام ، يوتيوب) والمواقع الإلكترونية للحصول على معلومات عن لقاح فيروس كورونا؟ | 1. نعم 2. لا   98. لا أعرف | |
| B6 | إذا نعم، ما المنصات التي تستخدمها؟ | 1. فيسبوك 2. تويتر 3. إنستجرام 4. يوتيوب 5. أخرى (حدد-----------------------) | |
| B7 | هل يدعم القادة (الدينيون، والسياسيون، والمعلمون، والعاملون في مجال الرعاية الصحية) في مجتمعك لقاحات كوفيد 19؟ | 1. نعم 2. البعض يعمل، والاخرين لا يعملون 3. لا 4. لا أعلم | |
| B8 | إذا نعم، هل توافقهم؟ | 1. نعم 2. احيانا 3. لا   98. لا أعلم | |
| B9 | هل تشعر أن لديك معلومات كافية عن لقاح كوفيد 19؟ | 1. نعم 2. احيانا 3. لا   98. لا أعلم | |
| B10 | هل أغلبية معارفك يرغبون أخذ تطعيم كوفيد 19؟ | 1. نعم 2. احيانا 3. لا   98. لا أعلم | |
|  | **تصور المجتمع** |  | |
| C1 | هل تشارك المعلومات المتعلقة بكوفيد 19 داخل شبكة التواصل الإجتماعي الخاصة بك؟ | 1. نعم 2. لا 3. NA | |
| C2 | إذا نعم، ماهي المعلومات؟ (إختر كل ما يمكن تطبيقة) | 1. توافر اللقاح 2. مخاوف بشأن الآثار الجانبية 3. معلومات حول الفائدة 4. عدم تشجيع الآخرين عن أخذه 5. تشجيع الآخرين عن أخذه 6. تسجيل نفسك 7. غير ذلك (حدد----------------) | |
| C3 | عندما تحصل على معلومات حول لقاح COVID-19 عبر وسائل التواصل الاجتماعي ، كيف تعرف ما إذا كان صحيحا؟ | 1. أتحقق من موقع ويب حسن السمعة؟ 2. أتحقق من خلال مقدم الرعاية الصحية 3. أثق في المعلومات التي أتلقاها مهما كانت 4. اطلب من المرسل توفير المصدر 5. لا أتحقق 6. غير ذلك (تحديد----------------------) | |
| C4 | كم عدد الأشخاص في المجتمع الذين تعتقد أنهم قلقون بشأن انتشار COVID-19 في المجتمع تقريبًا؟ تصور المجتمع | 1. معظم الناس 2. بعض الناس - أكثر من النصف 3. بعض الناس - أقل من النصف 4. قلة من الناس | |
| C5 | هل تعتقد أن لقاحات COVID-19 ستطرح أو يتم طرحها بشكل منصف لمجتمعك المحدد؟ | 1. نعم 2. لا   98. لا أعرف | |
|  | **ملاءمة، مناسب** |  | |
| D1 | هل ستحصل / حصلت على اللقاح إذا تم تقديمه في: (حدد كل ما ينطبق( | 1. مولات 2. دور عبادة (كنائس ، مساجد ، إلخ) 3. مكان العمل 4. لا يوجد مما سبق | |
| D2 | هل تفضل الحصول على / الحصول على اللقاح إذا تم تقديمه في (اختر واحدا) | 1. وحدات التطعيم المتنفلة 2. مراكز رعاية صحية أولية 3. مستشفيات 4. لا يوجد تفضيل | |
| D3 | **ما العوائق التي تواجهها (أو تتوقعها) لتلقي لقاح COVID-19؟** | 1. التوافر 2. المسافة إلى نقطة التطعيم 3. التكلفة 4. مسؤوليات أخرى 5. ليست مجموعة ذات أولوية 6. نقص المعلومات حول كيفية / مكان الحصول عليها 7. موقف الموظفين 8. غير مقبول اجتماعيا 9. مرهقة للغاية 10. لا شئ 11. غير ذلك (يرجى التحديد--------------) | |
|  | **الثقة** | |  |
| E1 | هل تعتقد أن اللقاح سيحميك من الإصابة كوفيد 19  **عدم الإجابة للذين تلقوا التطعيم** | 1. نعم 2. لا 3. بعض الشئ | |
| E2 | هل ستحصل / حصلت على اللقاح إذا أوصى صاحب العمل بذلك؟ | 1. نعم 2. لا 3. غير متأكد | |
| E3 | إذا لم يتم أخذها بالفعل ، فهل ستحصل على COVID-19 إذا رأيت أن جيرانك وقادة المجتمع والزعماء الدينيين والأطباء والمشاهير والسياسيين وما إلى ذلك قد تلقوا اللقاح بالفعل؟  **عدم الإجابة للذين تلقوا التطعيم** | 1. نعم 2. لا 3. لا أعلم | |
| E4 | هل تتذكر أي أحداث في الماضي من شأنها أن تثبط عزيمتك عن الحصول على لقاح COVID-19؟ | 1. نعم 2. لا | |
| E5 | هل تعتبر لقاح كوفيد 19 أمن؟ | 1. نعم 2. لا | |
| E5a | إذا نعم، أوصف | 1. خبرة سلبية مع اللقاح 2. عرفت شخص كان لدية تجربة سلبية مع اللقاح 3. لدية تجربة سلبية مع العاملين في مجال الرعاية الصحية\ المرافق الصحية 4. أخرى (حدد---------------------------) | |
| E6 | هل سبق أن تعرض شخص ما تعرفه لرد فعل سلبي خطير تجاه لقاح مما يجعلك مترددًا في الحصول على لقاح COVID-19 | 1. نعم 2. بعض اشئ 3. لا   98. لا أعلم | |
| E7 | هل تعتبر لقاح كوفيد 19 أمن؟ | 1. نعم 2. بعض اشئ 3. لا   98. لا أعلم | |
| E8 | هل أنت قلق بشأن أي مخاطر أو آثار جانبية للقاح COVID-19 | 1. نعم 2. بعض الشئ 3. لا   98. لا أعلم | |
| E8a | إذا نعم أو قليلا، ما نوع الخطر أو الأثار الجانبية | 1. حمى 2. آلام في الجسم 3. العقم 4. إعاقة جسدية 5. الموت 6. أخرى (حدد-------------) | |
| E9 | هل يوجد نوع من لقاح كوفيد 19 تفضلة؟ | 1. فايزر\بيونتك 2. مودرنا 3. أسترا زينيكا اكسفورد 4. جونسون جونسون 5. نوفا فاكس 6. سينوفاك 7. سبنوفارم 8. سبوتنك 9. كان سينو بيو 10. لا توجد تفضبلات 11. غير متأكد 12. أخرى (حدد--------------------) | |
| E10 | هل تعتقد أن هناك طرقًا أخرى (أفضل) للوقاية من COVID-19 بدلاً من اللقاح؟  إذا لا أو لا أعلم، انتقل الى E11 | 1. نعم 2. بعض الشئ 3. لا   98. لا أعلم | |
| E10a | إذا نعم أو بعض الشئ، ماهي هذه الطرق (إختر كل ما ينطبق) | 1. التباعد الاجتماعي 2. غسل اليدين 3. منع العدوى ومكافحتها 4. التهوية 5. لبس أقنعة الوجه 6. غير ذلك (حدد--------------) | |
| E11 | هل تثق بمقدمي الرعاية الصحية وموظفي الصحة المجتمعية لتزويدك بمعلومات دقيقة حول لقاح كوفيد 19؟ | 1. نعم 2. لا 3. بعض الشئ   98. لا أعلم | |
| E12 | هل تعتقد أن نظامك الصحي يمكنه إعطاء لقاح COVID-19 بأمان للسكان؟ | 1. نعم 2. لا 3. بعض الشئ   98. لا أعلم | |
|  | **الرضا عن النفس** |  | |
| F1 | هل تعتقد أنك معرض لخطر الإصابة بـ COVID-19 | 1. نعم 2. لا 3. بعض الشئ   98. لا أعلم | |
| F2 | هل تعتقد أنك يمكن أن تصاب بمرض خطير أو تدخل المستشفى أو تموت إذا أصبت بـ COVID-19؟ | 1. نعم 2. لا 3. بعض الشئ   98. لا أعلم | |
| F3 | هل تعرف أي شخص يعاني من مرض / إعاقة خطيرة بسبب عدم تلقيحهم؟ | 1. نعم 2. لا   98. لا أعلم | |
| F4 | هل تعتقد أنه من الأفضل الإصابة بـ COVID-19 وتطوير مناعة طبيعية بدلاً من الحصول على اللقاح؟ | 1. نعم 2. لا 3. بعض الشئ   98. لا أعلم | |
|  | **النوايا** |  | |
| G1 | هل ستؤخر تطعيم نفسك بلقاح تم تقديمه / موصى به حديثًا؟  **عدم الإجابة للذين تلقوا التطعيم** | 1. نعم 2. لا 3. 98. لا أعلم | |
| G2 | إذا كان اللقاح متاحًا ، فهل ستحصل عليه؟ (إذا حصلت على اللقاح ، فانتقل إلى النهاية)  **عدم الإجابة للذين تلقوا التطعيم** | 1. نعم 2. لا   98. غيرمتأكد | |
| G3 | هل لديك أي تعليقات أو ملاحظات حول لقاح COVID-19 تود مشاركتها معنا؟ |  | |

**شكرا لك على المشاركة في هذه الدراسة!**

تعليقات مجري المقابلة

-----------------------------------------------------------------------------------------------------------------------------------------------------------------------------------------------------------------------------

**S1.b. Vaccine Hesitancy Assessment Qualitative Discussion Guide**

| **Enumerator/Interviewer Name** |  |
| --- | --- |
| **Location^[[5]](#footnote-5)^:** |  |
| **FDG or KII** |  |
| **Date:** |  |
| **Time of Interview:** |  |
| **Project name^[[6]](#footnote-6)^** |  |
| **READ:** Hello I am working on a study for IMC XXX^[[7]](#footnote-7)^. The purpose of the study is to collect information to better understand perceptions of community members and health care workers on COVID-19 vaccines, and to assess interest in COVID-19 vaccination and reasons for hesitancy and concerns. I would like to ask you some questions about your perception of the COVID-19 vaccines. Your answers are confidential and cannot be linked back to you. Your participation is completely voluntary and you may decline to answer any specific question or completely refuse to participate. The interview should take about 30 minutes of your time and you will not be contacted in the future. We would greatly appreciate your help in responding to these questions. | |
| **Are you willing to be interviewed at this time?** | ☐ 1. Yes  ☐ 2. No |

**Thematic area: Perception and Information**

**Q. 1 Can you let me know what you think of the COVID-19 vaccines and where do you get information from?**

**Probing questions**: - (Note: - probing questions are asked if the responder(s) does not cover any of the following points; this is not a questionnaire instead it is an interview guide and based on the responses the interviewer can add additional questions that would help clarify the point(s) raised by the respondent)

- What do you think about the vaccine?
- Do you think that it safe for you to take it if offered?
- Do you think that one vaccine is more safe than the other, and which one due you think is safe?
- Would you take it if offered today?
- Is there a type of COVID vaccine that you would prefer and why?
- Where do you get information on the vaccine?
- Which source of information do you trust most? Which source of information you do not trust?
- When you get information on the vaccine, how do you know it is accurate or not?

**Thematic area: - enablers**

**Q. 2 What would encourage you to take the vaccine?**

**Probing questions**: -

- Get more accurate information about the vaccine
- Seeing community leaders or influential people, HCW, your neighbor or someone you know take the vaccines?
- If offered a specific type of COVID vaccine, which one?

**Thematic area: barriers**

**Q. 3 What is preventing you from taking the vaccine?**

**Probing questions: -**

- Lack of information about the vaccine
- Rumors and myths about the vaccine, please specify what type of rumors and myths you are hearing
- Are you concerned about any risks or side effects with this vaccine? What kind of risks or side effects are you concerned about?
- Any bad past experience with vaccination (either COVID or any other vaccine), with you or someone you know
- Do you think it is better to get COVID-19 and develop natural immunity than to get the vaccine?

**Thematic area: Effect of Conflict on Perceptions of COVID-19 in Gaza**

**Q. 4 KAP before and after the conflict**

- Did you practice any of these preventive measures before the conflict? If yes, what were they?
- Probe: Masks? Social distancing? Hand washing?
- Probe: Did family and friends/ colleagues practice these measures?
- Probe: Did your community practice these measures?
- Did you continue practicing these measures during the conflict? If not, why not?
- Probe: Masks? Social distancing? Hand washing?
- Probe: Did family and friends/ colleagues practice these measures?
- Probe: Did your community practice these measures?
- Have your perceptions and practices of these preventive measures changed after the conflict? Why or why not (probably the same reasons as above)?
- Probe: Masks? Social distancing? Hand washing?
- Probe: Did family and friends/ colleagues practice these measures?
- Probe: Did your community practice these measures?
- There may be people you know of (in your community/ work) that do not wear masks or practice social distancing. Why do you think they may not/ will not wear masks or practice social distancing?
- Probe: Did their practice change before or after the conflict?
- Probe: Due to knowledge and perceptions?
- Probe: Due to cultural and religious issues?
- Probe: If not practicing, would you start practicing if you saw other community members practicing?
- What are the three main barriers that people faced in regularly implementing COVID-19 related prevention measures during and after the conflict, and can you think of any solutions to these?

| Barrier | Solution |
| --- | --- |
| 1. |  |
| 2. |  |
| 3. |  |

- Do you think COVID-19 is still a big problem in Gaza after the conflict? Why or why not?
- Do you know if the MOH measures for reducing the spread of COVID-19 are still in place after the conflict? Which ones? To what degree?
- Probe: Has the intensity of the MOH’s messaging increased/ reduced?

**Q. 5 Concluding question: - do you want to share any other information that we have not covered so far?**

Annex 4: دليل المقابلة

|  | **اسم مجري المقابلة أو رقمه** |
| --- | --- |
|  | **الموقع** |
|  | **أسم الشخص** |
|  | **التاريخ** |
|  | **وقت المقابلة** |
|  | **اسم المشروع أو البلد** |
| السلام عليكم، أنا أعمل على دراسة عن التردد في التطعيم ضد كوفيد 19. الغرض من الدراسة هو جمع المعلومات لفهم تصورات أفراد المجتمع والعاملين في مجال الرعاية الصحية حول لقاحات COVID-19 بشكل أفضل ، وتقييم الاهتمام بتلقيح COVID-19 وأسباب التردد والمخاوف. أود أن أطرح عليك بعض الأسئلة حول تصورك للقاحات COVID-19. إجاباتك سرية ولا يمكن ربطها بك. مشاركتك تطوعية تمامًا ويمكنك رفض الإجابة على أي سؤال محدد أو رفض المشاركة تمامًا. يجب أن تستغرق المقابلة حوالي 30 دقيقة من وقتك ولن يتم الاتصال بك في المستقبل. سنكون ممتنين لمساعدتكم في الرد على هذه الأسئلة | |
| 1. **نعم** 2. **لا** | **هل ترغب في المقابلة الأن** |

**المجال المواضيعي: التصور والمعلومات**

س 1: هل يمكنك إخباري برأيك في لقاحات COVID-19 ومن أين تحصل على المعلومات؟

1. ما رأيك في اللقاح؟
2. هل تعتقد أنه آمن لك تناولها إذا عرضت عليك؟
3. هل تعتقد أن أحد اللقاحات أكثر أمانًا من الآخر ، وأي لقاح تعتقد أنه أمن؟
4. هل ستأخذه إذا عرض عليه اليوم؟
5. هل هناك نوع من لقاح COVID تفضله ولماذا؟
6. من أين تحصل على معلومات عن اللقاح؟
7. ما هو مصدر المعلومات الذي تثق به أكثر من غيره؟ ما هو مصدر المعلومات الذي لا تثق به؟
8. عندما تحصل على معلومات عن اللقاح ، كيف تعرف أنه دقيق أم لا؟

**المجال المواضيعي: - عوامل التمكين**

س 2: ما الذي يشجعك على أخذ اللقاح؟

أسئلة استقصائية:-

1. احصل على معلومات أكثر دقة حول اللقاح
2. رؤية قادة المجتمع أو الأشخاص المؤثرين، HCW، أو جارك أو شخص تعرفه يأخذ اللقاحات؟
3. في حالة تقديم نوع معين من لقاح COVID ما هو؟

**المجال المواضيعي: الحواجز**

س 3: ما الذي يمنعك من أخذ اللقاح؟

أسئلة استقصائية:-

1. نقص المعلومات حول اللقاح
2. شائعات وأساطير حول اللقاح ، يرجى تحديد نوع الشائعات والأساطير التي تسمها
3. هل أنت قلق من أي مخاطر أو آثار جانبية لهذا اللقاح؟ ما نوع المخاطر أو الآثار الجانبية التي تشعر بالقلق حيالها؟
4. أي تجربة سابقة سيئة مع التطعيم (إما COVID أو أي لقاح آخر) ، معك أو مع أي شخص تعرفة
5. هل تعتقد أنه من الأفضل الإصابة بـ COVID-19 وتطوير مناعة طبيعية بدلاً من الحصول على اللقاح؟

**المجال المواضيعي: تأثير الصراع على تصورات COVID-19 في غزة**

س 4: المعرفة، المواقف والمهارات قبل وبعد الصراع

هل مارست أيًا من هذه الإجراءات الوقائية قبل النزاع؟ إذا كانت الإجابة بنعم ، فماذا كانت

- دقق: أقنعة؟ الإبعاد الاجتماعي؟ غسل اليدين؟
- دقق: هل مارس أفراد العائلة والأصدقاء / الزملاء هذه الإجراءات

هل استمريت في ممارسة هذه الإجراءات أثناء النزاع؟ إذا لم يكن كذلك ، فلماذا؟

- دقق: أقنعة؟ الإبعاد الاجتماعي؟ غسل اليدين؟
- دقق: هل مارس أفراد العائلة والأصدقاء / الزملاء هذه الإجراءات؟
- دقق: هل مارس أفراد العائلة والأصدقاء / الزملاء هذه الإجراءات؟

هل تغيرت تصوراتك وممارساتك بشأن هذه التدابير الوقائية بعد الصراع؟ لماذا أو لماذا لا (ربما نفس الأسباب المذكورة أعلاه)؟

• دقق: أقنعة؟ الإبعاد الاجتماعي؟ غسل اليدين؟

• دقق: هل مارس أفراد العائلة والأصدقاء / الزملاء هذه الإجراءات؟

• دقق: هل مارس مجتمعك هذه الإجراءات؟

قد يكون هناك أشخاص تعرفهم (في مجتمعك / عملك) لا يرتدون أقنعة أو يمارسون التباعد الاجتماعي. لماذا تعتقد أنهم قد لا يرتدون الأقنعة أو لا يمارسون التباعد الاجتماعي؟

• دقق: هل تغيرت ممارساتهم قبل الصراع أو بعده؟

• دقق: بسبب المعرفة والتصورات؟

• دقق: بسبب القضايا الثقافية والدينية؟

• دقق: إذا لم تكن تمارس ، فهل ستبدأ التدرب إذا رأيت أعضاء آخرين في المجتمع يتدربون؟

ما هي العوائق الرئيسية الثلاثة التي واجهها الأشخاص في التنفيذ المنتظم لتدابير الوقاية المتعلقة بـ COVID-19 أثناء النزاع وبعده ، وهل يمكنك التفكير في أي حلول لهذة؟

| الحلول | الحواجز |
| --- | --- |
|  | 1. |
|  | 2. |
|  | 3. |

هل تعتقد أن COVID- لا يزال يمثل مشكلة كبيرة في غزة بعد الصراع؟ لما و لما لا؟

هل تعرف ما إذا كانت إجراءات وزارة الصحة للحد من انتشار COVID-19 لا تزال سارية بعد الصراع؟ اي واحدة؟ إلى أي درجة؟

• التحقيق: هل زادت / انخفضت كثافة رسائل وزارة الصحة؟

س 5: السؤال الختامي: - هل ترغبون في مشاركة أي معلومات أخرى لم نقم بتغطيتها حتى الآن؟

1. Country teams can add choices for location to standardize this (especially if doing digital data collection) [↑](#footnote-ref-1)
2. Only if doing a cluster survey; if country teams implementing systematic or simple random sample, take this out [↑](#footnote-ref-2)
3. Insert name of the country or project (project if this survey is attached to some project) based on the appropriateness [↑](#footnote-ref-3)
4. Might want to reduce these questions to the ones where you think there will be meaningful heterogeneity in answers (keeping in mind that we are powering the survey for overall population) [↑](#footnote-ref-4)
5. Country teams can add choices for location to standardize this (especially if doing digital data collection) [↑](#footnote-ref-5)
6. Insert name of the country or project (project if this survey is attached to some project) based on the appropriateness [↑](#footnote-ref-6)
7. [↑](#footnote-ref-7)
