# Supplementary figures and images for: COVID-19 vaccination in the Gaza Strip: a cross-sectional study of vaccine coverage, hesitancy, and associated risk factors among community members and healthcare workers
Source: Confl Health. 2022 Sep 9;16:48. doi: 10.1186/s13031-022-00477-7 (PMC9461392; doi:10.1186/s13031-022-00477-7)

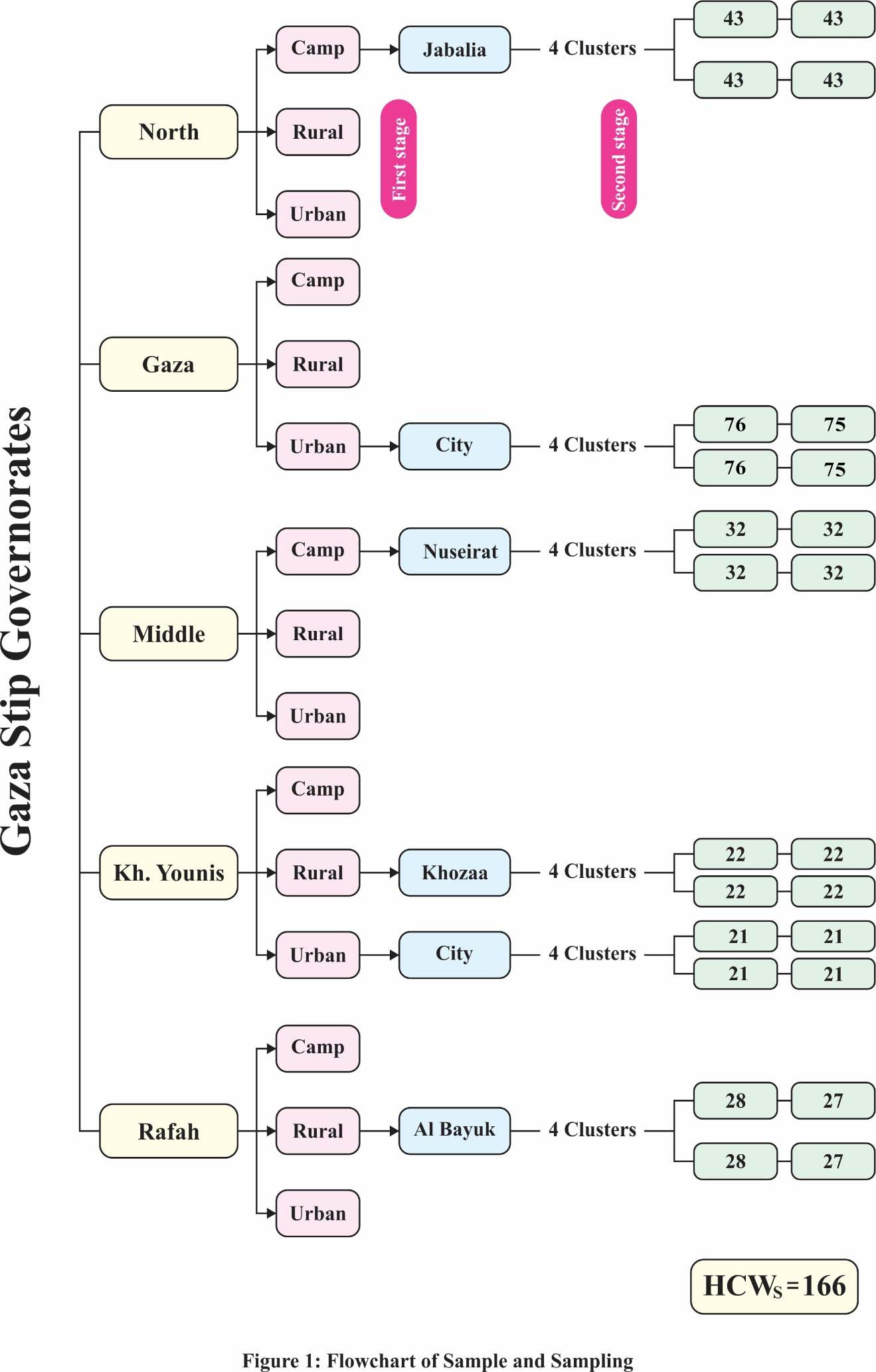

Supplement: Supplementary file 2 — Additional file 2. Flow chart of multi-stage sampling approach used for community survey. [file 13031_2022_477_MOESM2_ESM.docx]
