# Supplementary material for: COVID-19 vaccination in the Gaza Strip: a cross-sectional study of vaccine coverage, hesitancy, and associated risk factors among community members and healthcare workers
Source: Confl Health. 2022 Sep 9;16:48. doi: 10.1186/s13031-022-00477-7 (PMC9461392; doi:10.1186/s13031-022-00477-7)
Supplement: Supplementary file 4 — Additional file 4. Finite population correction (FPC) and sampling weights per cluster. [file 13031_2022_477_MOESM4_ESM.docx]

Finite population correction (FPC) and probability of selection for stages of multi-stage sampling

| **Geo-location** | **Cluster** | **Strata** | **Geo-location** | | **Cluster** | | **Household** | | | **Sampling weight** |
| --- | --- | --- | --- | --- | --- | --- | --- | --- | --- | --- |
|  |  |  | **Probability of selection** | **FPC** | **Probability of selection** | **FPC** | **Probability of selection** | **Number in the sample** | **FPC** |  |
| GAZA CITY | G3 | Urban | 0.1176471 | 17 | 0.008988764 | 445 | 0.21189334 | 53 | 250 | 2.51355374 |
|  | G4 | Urban | 0.1176471 | 17 | 0.008988764 | 445 | 0.21189334 | 53 | 250 | 2.51355374 |
|  | GC1 | Urban | 0.1176471 | 17 | 0.008988764 | 445 | 0.35582089 | 89 | 250 | 1.49683538 |
|  | GC2 | Urban | 0.1176471 | 17 | 0.008988764 | 445 | 0.47176253 | 118 | 250 | 1.12896905 |
| JABALIA | J1 | Camp | 0.2500000 | 4 | 0.102564103 | 39 | 0.19022416 | 47 | 247 | 0.11547433 |
|  | J2 | Camp | 0.2500000 | 4 | 0.102564103 | 39 | 0.23069738 | 57 | 247 | 0.09521568 |
|  | J3 | Camp | 0.2500000 | 4 | 0.102564103 | 39 | 0.16998755 | 42 | 247 | 0.12922128 |
|  | J4 | Camp | 0.2500000 | 4 | 0.102564103 | 39 | 0.17808219 | 44 | 247 | 0.12334758 |
| KHANYUNIS | K1 | Rural | 0.2500000 | 4 | 0.444444444 | 9 | 0.09032847 | 22 | 244 | 0.05611829 |
|  | K2 | Rural | 0.2500000 | 4 | 0.444444444 | 9 | 0.09032847 | 22 | 244 | 0.05611829 |
|  | K3 | Urban | 0.1176471 | 17 | 0.025316456 | 158 | 0.08804904 | 22 | 250 | 2.14772133 |
|  | K4 | Rural | 0.2500000 | 4 | 0.444444444 | 9 | 0.08622263 | 21 | 244 | 0.05879059 |
| KHANYUNIS | KY1 | Urban | 0.1176471 | 17 | 0.025316456 | 158 | 0.09205127 | 23 | 250 | 2.05434215 |
|  | KY2 | Urban | 0.1176471 | 17 | 0.025316456 | 158 | 0.08004458 | 20 | 250 | 2.36249347 |
|  | KY3 | Urban | 0.1176471 | 17 | 0.025316456 | 158 | 0.08804904 | 22 | 250 | 2.14772133 |
|  | KY4 | Urban | 0.1176471 | 17 | 0.025316456 | 158 | 0.08404681 | 21 | 250 | 2.24999378 |
| NUSEIRAT | N1 | Camp | 0.2500000 | 4 | 0.166666667 | 24 | 0.13463125 | 34 | 253 | 0.10040421 |
|  | N2 | Camp | 0.2500000 | 4 | 0.166666667 | 24 | 0.13859099 | 35 | 253 | 0.09753551 |
|  | N3 | Camp | 0.2500000 | 4 | 0.166666667 | 24 | 0.07523511 | 19 | 253 | 0.17967068 |
|  | N4 | Camp | 0.2500000 | 4 | 0.166666667 | 24 | 0.17818842 | 45 | 253 | 0.07586095 |
| RAFAH | R1 | Rural | 0.2500000 | 4 | 0.022346369 | 179 | 0.05359975 | 13 | 243 | 1.88094819 |
|  | R2 | Rural | 0.2500000 | 4 | 0.022346369 | 179 | 0.04947669 | 12 | 243 | 2.03769387 |
|  | R3 | Rural | 0.2500000 | 4 | 0.022346369 | 179 | 0.14430701 | 35 | 243 | 0.69863790 |
|  | R4 | Rural | 0.2500000 | 4 | 0.022346369 | 179 | 0.15255312 | 37 | 243 | 0.66087369 |
